# STK38L kinase ablation promotes loss of cell viability in a subset of KRAS-dependent pancreatic cancer cell lines

**DOI:** 10.18632/oncotarget.20833

**Published:** 2017-09-11

**Authors:** Trevor J. Grant, Anita K. Mehta, Anamika Gupta, Ahmad A.D. Sharif, Kshitij S. Arora, Vikram Deshpande, David T. Ting, Nabeel Bardeesy, Neil J. Ganem, Alexander Hergovich, Anurag Singh

**Affiliations:** ^1^ Department of Pharmacology and Experimental Therapeutics, Center for Cancer Research, Boston University Graduate School of Medicine, Boston, MA, USA; ^2^ University College London, Cancer Institute, London, United Kingdom; ^3^ Massachusetts General Hospital Cancer Center, Harvard Medical School, Charlestown, MA, USA

**Keywords:** KRAS, oncogene dependency, Hippo pathway, pancreatic cancer, kinase

## Abstract

Pancreatic ductal adenocarcinomas (PDACs) are highly aggressive malignancies, associated with poor clinical prognosis and limited therapeutic options. Oncogenic *KRAS* mutations are found in over 90% of PDACs, playing a central role in tumor progression. Global gene expression profiling of PDAC reveals 3-4 major molecular subtypes with distinct phenotypic traits and pharmacological vulnerabilities, including variations in oncogenic KRAS pathway dependencies. PDAC cell lines of the aberrantly differentiated endocrine exocrine (ADEX) subtype are robustly KRAS-dependent for survival. The *KRAS* gene is located on chromosome 12p11-12p12, a region amplified in 5-10% of primary PDACs. Within this amplicon, we identified co-amplification of *KRAS* with the *STK38L* gene in a subset of primary human PDACs and PDAC cell lines. Therefore, we determined whether PDAC cell lines are dependent on *STK38L* expression for proliferation and viability. *STK38L* encodes a serine/threonine kinase, which shares homology with Hippo pathway kinases LATS1/2. We show that *STK38L* expression is elevated in a subset of primary PDACs and PDAC cell lines displaying ADEX subtype characteristics, including overexpression of mutant KRAS. RNAi-mediated depletion of STK38L in a subset of ADEX subtype cell lines inhibits cellular proliferation and induces apoptosis. Concomitant with these effects, STK38L depletion causes increased expression of the LATS2 kinase and the cell cycle regulator p21. LATS2 depletion partially rescues the cytostatic and cytotoxic effects of STK38L depletion. Lastly, high *STK38L* mRNA expression is associated with decreased overall patient survival in PDACs. Collectively, our findings implicate STK38L as a candidate targetable vulnerability in a subset of molecularly-defined PDACs.

## INTRODUCTION

Pancreatic ductal adenocarcinoma (PDAC) is typically associated with poor clinical prognosis and is the fourth leading cause of cancer-related deaths in the United States [1]. At present, conventional chemotherapies used to treat PDAC, such as 5-fluorouracil and gemcitabine, fail to improve patient survival beyond several weeks, underscoring the need to develop more effective therapeutic agents [2]. A critical barrier to the development of selective and efficacious therapeutics for the treatment of PDAC is the heterogeneous nature of the disease. Oncogenic *KRAS* mutations are found in over 90% of PDACs. Co-occurring alterations in *CDKN2A*/p16, *TP53*, and *SMAD4/DPC4* tumor suppressor genes are also prevalent at high frequencies [3, 4]. Despite the presence of these common genetic alterations, PDACs exhibit a high degree of inter- and intratumoral molecular and histological heterogeneity. Whole genome transcriptional profiling reveals 3-4 PDAC molecular subtypes that are associated with distinct phenotypic traits and pharmacological vulnerabilities [4, 5]. In order to develop more selective therapeutic modalities to treat PDAC, it is critical to understand the differences in activated signaling networks when comparing these contrasting subtypes.

The “aberrantly differentiated endocrine exocrine-like” (ADEX) PDAC subtype is characterized by high expression of epithelial differentiation genes and overexpression of oncogenic *KRAS*. These features are found in a subset of *KRAS*-mutant PDAC cell lines that exhibit robust dependence on KRAS for survival [6]. KRAS-dependent cells frequently harbor *KRAS* gene amplification and undergo apoptosis following KRAS depletion, indicating a state of *KRAS* oncogene “addiction” or dependency. Indeed, KRAS plays a critical role in PDAC initiation and maintenance, making it an attractive therapeutic target [7]. However, attempts to develop KRAS-directed therapies for clinical use have proven challenging [8–10]. This obstacle has prompted the search for alternative therapeutic targets to treat PDAC by identifying synthetic lethal *KRAS* interacting genes that confer a state of non-oncogene dependency [11]. We previously identified non-oncogene dependency for the nuclear Dbf2 and LATS1/2-related kinase STK38L (also known as NDR2) in the *KRAS*-mutant SW620 colon cancer cell line [12]. Subsequently, we noted that the *STK38L* and *KRAS* genes are located in close proximity on chromosome 12p11-12, a region frequently amplified in solid tumors, including those of the colon and pancreas [13–16]. Oncogene amplification is often related to oncogene “addiction/dependency,” as is the case for the *MYC* oncogene [17]. This provided rationale to investigate whether *STK38L* gene copy number gain correlated with STK38L dependency on a larger scale in PDAC cell lines and to ultimately determine the potential of STK38L as a candidate therapeutic target. STK38L can play context-dependent, oncogenic or tumor suppressive roles. STK38L-mediated tumor suppression can occur via promotion of YAP phosphorylation and regulation of the Hippo signaling pathway [18]. Oncogenic functions of STK38L include regulation of MYC protein stability [19]. Additionally, STK38L regulates the stability of the CDK inhibitor p21 via direct phosphorylation [20]. Many of these functions of STK38L have been attributed to the closely related isoform STK38, also known as NDR1, although distinct STK38L-specific non-redundant functions are likely to exist [21]. Therefore, it is important to determine context-dependent STK38L-specific signaling mechanisms in PDAC cell lines.

Here, we assessed *STK38L* gene amplification as well as mRNA and protein expression in a panel of human PDAC cell lines and primary tumors. We determined correlative relationships comparing *STK38L* with *KRAS* gene amplification and expression. We investigated the role of STK38L in promoting proliferation and viability in PDAC cell lines. To identify potential mechanisms of STK38L-dependent survival signaling, we investigated downstream consequences of STK38L depletion in PDAC cell lines on LATS1/2 and p21 protein expression and function. Finally, we analyzed *STK38L* mRNA expression patterns in primary tumors from PDAC patients and correlated *STK38L* expression with overall survival.

## RESULTS

### STK38L gene copy number and protein expression levels are elevated in subsets of human PDAC

**Figure 1:**
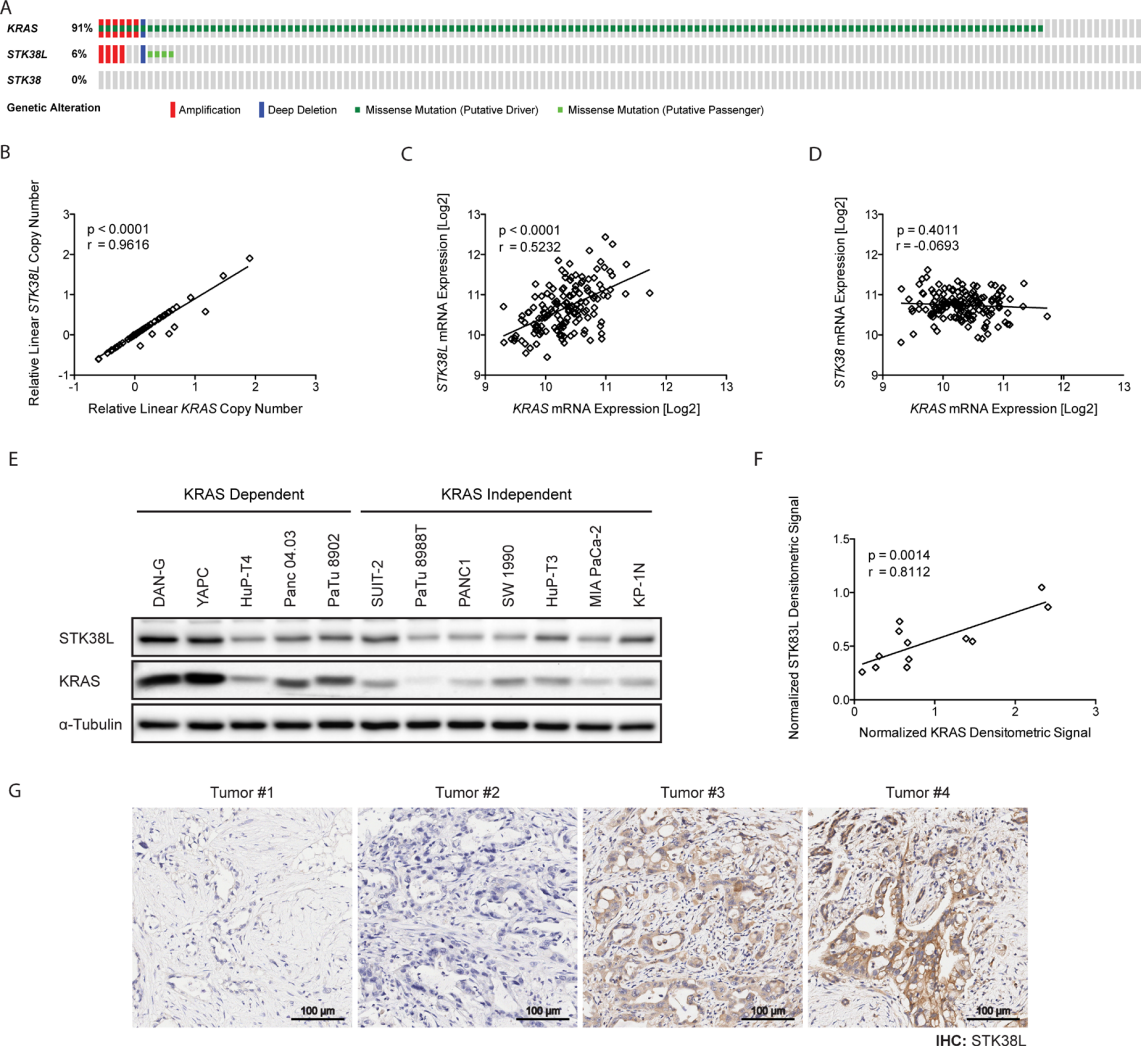
*STK38L* and *KRAS* expression levels correlate in primary PDACs and PDAC-derived cell lines (**A**) Oncoprint summary of *KRAS*, *STK38*, and *STK38L* genomic alterations in the TCGA PDAC cohort. Individual genes are represented by rows, and individual tumors are represented by columns. (**B**) Correlation analysis of *STK38L* and *KRAS* relative linear copy number values. (**C**) Correlation analysis of *STK38L* and *KRAS* mRNA expression (RNA-Seq V2 RSEM). (**D**) Correlation analysis of *STK38* and *KRAS* mRNA expression (RNA-Seq V2 RSEM) (**E**) Western blot of STK38L and KRAS protein levels in human-derived PDAC cell lines. Data are representative of two independent experiments. (**F**) Correlation analysis of STK38L and KRAS protein expression in human-derived PDAC cell lines. (**G**) Representative images of STK38L immunohistochemical staining (brown) in human PDAC tissue samples at 20× magnification. Cells are counterstained with haematoxylin (blue). Scale bar = 100 µm.

To assess the prevalence of *STK38L* gene copy number alterations in human PDAC, we analyzed the pancreatic adenocarcinoma (PAAD) dataset from The Cancer Genome Atlas (TCGA) (Figure 1A). Consistent with previous studies, *KRAS* mutations are found in over 90% of cases in the TCGA PDAC cohort. *STK38L* gene amplification was observed in 2.7% percent of tumors. *STK38L* copy number gains were concordant with *KRAS* gene amplification. However, two tumors exhibited *KRAS* amplification alone. Using TCGA PDAC SNP and RNA-seq-based gene expression data, we performed a linear regression analysis and observed a positive correlation (*r* = 0.9616; *p* < 0.0001) between *KRAS* and *STK38L* gene copy number values (Figure 1B). We also observed a positive correlation between *STK38L* and *KRAS* mRNA expression (*r* = 0.5232; *p* < 0.0001) (Figure 1C). In contrast, we failed to observe a significant correlation between expression levels of the *STK38* isoform and *KRAS* (*r* = −0.0693; *p* = 0.4011) (Figure 1D). Next, we performed correlation analyses of expression levels for other genes located within the chromosome 12p11-12 amplicon (*RASSF8, SSPN, FGFR1OP2, MED21, BHLHE41* and *ITPR2*). With the exception of *MED21, KRAS* and *STK38L* mRNA expression levels were the most correlated of all the genes tested (Supplementary Figure 1). These findings indicate that the correlation of *KRAS* and *STK38L* expression levels is not simply due to proximal genomic context and could be due to transcriptional co-regulation.

We subsequently assessed STK38L protein levels in a panel of twelve established and well characterized human PDAC-derived cell lines (Supplementary Table 1) by Western blotting (Figure 1E). STK38L protein expression varied considerably across the panel of cell lines. We also assessed KRAS protein levels in the cell line panel and observed a similar degree of variation, with some cell lines expressing high levels of KRAS protein, as previously reported [6]. Notably, we observed a significant correlation between STK38L and KRAS protein levels as determined by pixel densitometric analysis (*r* = 0.8122, *p* = 0.0014) (Figure 1F). We conclude that STK38L and KRAS gene copy number, mRNA, and protein levels are tightly correlated in human PDAC and tumor-derived cell lines.

To assess whether STK38L expression is elevated in primary PDACs, we analyzed a series of human PDAC tissue microarray (TMA) slides by immunohistochemistry (Figure 1G). STK38L protein levels varied significantly across the primary tumor cohort. A subset of tumors was found to express high levels of STK38L protein. In contrast, STK38L expression was weak or barely detectable in another subset of tumors. In summary, STK38L protein expression levels follow a variable spectrum both in cell lines and in primary PDACs.

### STK38L depletion promotes cell death in a subset of human-derived PDAC cell lines

**Figure 2:**
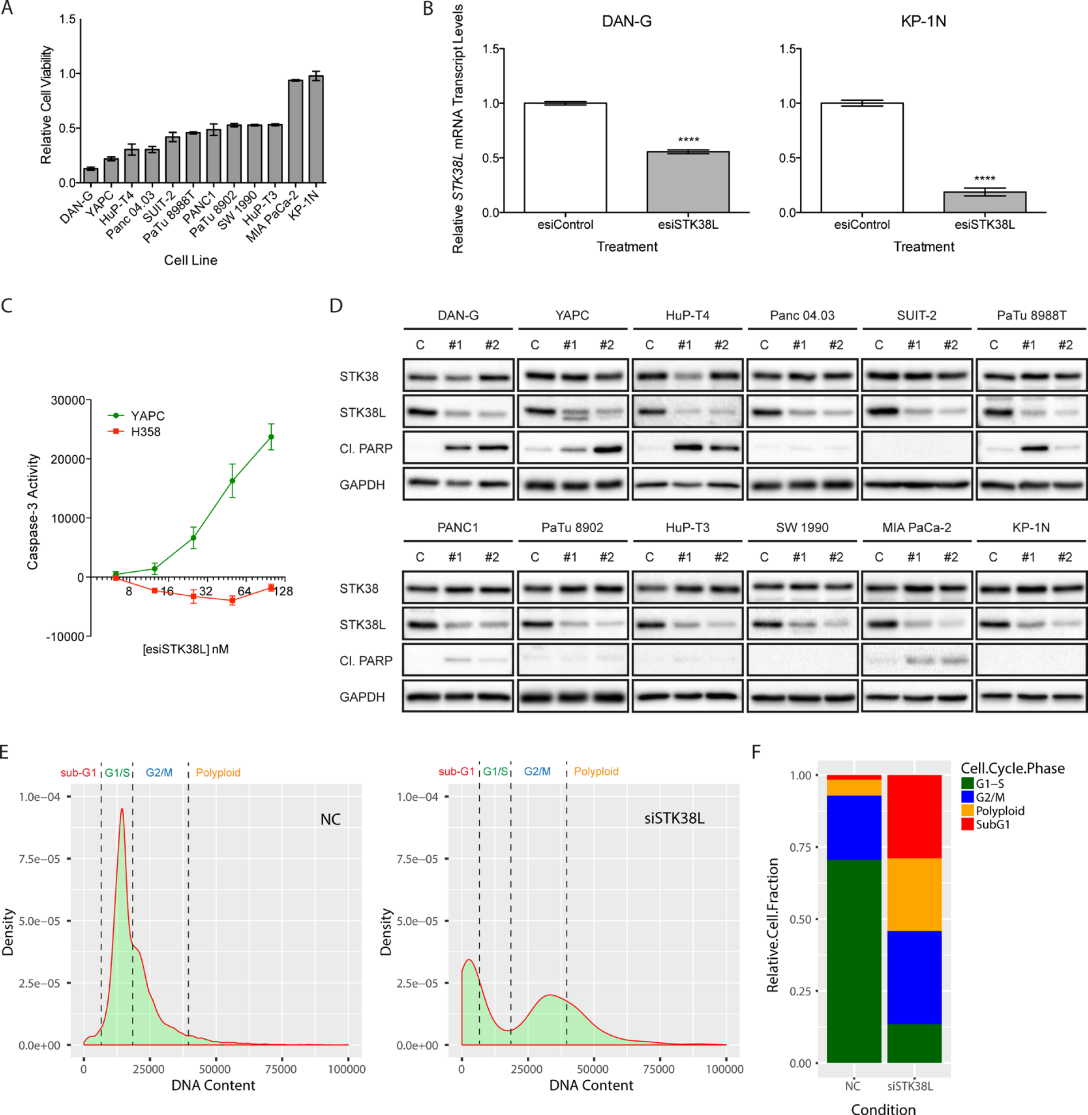
STK38L promotes cell survival in a subset of PDAC cell lines (**A**) Assessment of relative cell viability by Alamar Blue assay in a panel of PDAC cell lines 72 h post-transfection with pooled esiRNAs directed against *STK38L* at 25 nM final concentration. Values are normalized to cells transfected with control esiRNAs. Data are represented as the mean of six technical replicates +/− SEM. (**B**) RT-qPCR analysis of relative *STK38L* mRNA transcript levels for DAN-G and KP-1N cells 24 h post-transfection with pooled esiRNAs directed against *STK38L*. Transcript levels are normalized to the reference gene *GAPDH*. Data are the mean of three technical replicates +/− SEM. Statistical significance was assessed by Student’s *t*-test (****−p < 0.0001). (**C**) Luciferase-based caspase-3-Glo reporter assays of YAPC PDAC cells and H358 lung cancer cells transfected with increasing concentrations of esiSTK38L for 48 h. Arbitrary luminescence units, which are proportional to caspase-3 activity, are plotted on the vertical axis. Data are presented as the means of three replicates +/− SEM. (**D**) Western blots showing levels of cleaved PARP as an indicator of apoptosis in PDAC cell lines 48 h post-transfection with individual siRNA sequences (#1 and #2) directed against *STK38L*. GAPDH serves as a gel loading control. (**E**) Density histogram plots of image cytometry data showing cell counts based on nuclear DNA content, as measured by Hoechst staining, in NC control or siSTK38L transfected DAN-G cells. (**F**) Relative cell population quantitation based on cell cycle phase, derived by “gating” histograms shown in panel E. All data are representative of two or three independent experiments.

To determine the role of STK38L in promoting cell proliferation and survival in PDAC cell lines, we performed a series of functional experiments using *STK38L-*selective synthetic RNAi oligonucleotides or lentiviral-based shRNAs from various sources. First, we used pooled endoribonuclease-prepared small interfering RNAs (esiRNAs), which have been used effectively in genome-wide RNAi screens [22]. Transfection of *STK38L*-directed esiRNAs was performed to determine effects on relative cell proliferation and viability (Figure 2A). STK38L depletion caused a spectrum of growth inhibitory effects in a PDAC cell line panel. Of note, a subset of cell lines was highly sensitive to the growth inhibitory effects of STK38L depletion, including the DAN-G and YAPC cell lines. In contrast, MIA PaCa-2 and KP-1N were insensitive to *STK38L* depletion. *STK38L* mRNA knockdown was validated by qPCR in representative cell lines from opposite ends of the sensitivity spectrum, DAN-G and KP-1N (Figure 2B). We validated significant reduction in *STK38L* mRNA levels in both cell lines following esiRNA transfection. Importantly, the *STK38L-*directed esiRNAs did not display off-target effects on related *STK38*, *LATS1*, and *LATS2* mRNA expression (Supplementary Figure 2A). On the contrary, *LATS1* and *LATS2* levels were increased following *STK38L* depletion. To determine if esiRNA-mediated STK38L depletion caused apoptotic cell death, we employed a luminescence-based assay to measure levels of activated effector caspase-3 in cells following STK38L esiRNA transfection. In the YAPC PDAC cell line, we observed an esiRNA dose-dependent increase in activated caspase-3, indicating strong induction of apoptotic cell death following STK38L knockdown (Figure 2C). In contrast, STK38L knockdown failed to induce apoptosis in a *KRAS* mutant lung cancer cell line H358. Like YAPC cells, H358 cells are KRAS dependent. However, unlike YAPC cells, H358 cells do not exhibit increased *STK38L* gene copy number. Therefore, esiRNAs directed against STK38L selectively induce caspase-3 activation and apoptotic cell death in a PDAC cell line that harbors combined *KRAS* and *STK38L* gene amplification.

Next, we determined the effect of STK38L depletion on levels of cleaved poly(ADP-ribose) polymerase (PARP), which is a marker of caspase-3-mediated apoptosis (Figure 2D). For these assays, we used two STK38L-selective siRNA oligonucleotides. These siRNAs did not significantly affect protein levels of the STK38 isoform (Figure 2D). The three cell lines that were most sensitive to the growth inhibitory effects of STK38L-directed esiRNAs, DAN-G, YAPC, and HuP-T4, displayed robust induction of cleaved PARP in response to STK38L depletion with both siRNAs. Although Panc 04.03 cells were strongly growth inhibited following esiSTK38L introduction, we did not observe any PARP cleavage in these cells using single siRNAs. The remaining cell lines were relatively unresponsive to the effects of STK38L depletion on cleaved PARP levels. We validated these contrasting effects on PARP cleavage using STK38L-targeted esiRNAs in DAN-G and KP-1N cells (Supplementary Figure 2B). To rule out the possibility that KP-1N cells are generally resistant to apoptosis, we treated DAN-G and KP-1N cells with the apoptosis-inducing agent anisomycin, and showed comparable effects in both cell lines (Supplementary Figure 2C). Finally, we used image-based cytometry of Hoechst dye-stained cellular nuclei in DAN-G cells to quantitate relative effects of siSTK38L transfection on cell cycle phases, including non-viable sub-G1 phase cells (Figure 2E). Ablation of STK38L caused a robust increase in the proportion of sub-G1 cells (28% of the cell population) (Figure 2F). Furthermore, STK38L ablation caused reduced numbers of cells in G1/S phases, and an accumulation of cells in G2/M phases perhaps indicative of a block in mitosis (Figure 2F). Taken together, these findings suggest that selective knockdown of STK38L elicits a spectrum of antiproliferative effects in human PDAC cell lines, with a subset of cell lines undergoing PARP-cleavage associated apoptotic cell death.

Since oncogene dependency is often associated with copy number gain, we assessed *STK38L* gene copy number via genomic qPCR in PDAC cell lines (Supplementary Figure 3A). We observed varying *STK38L* gene copy number gains across the cell line panel. However, gene copy number failed to correlate (*r* = 0.4737; *p* = 0.1198) with the antiproliferative effects of STK38L depletion (Supplementary Figure 3B). We subsequently assessed *STK38L* mRNA transcript levels in the same cell line panel by qPCR (Supplementary Figure 3C). We observed a positive correlation (*r* = 0.8339; *p* = 0.0007) between *STK38L* transcript levels and STK38L dependency (Supplementary Figure 3D). Furthermore, the STK38L-dependent cell lines, DAN-G and YAPC, had significantly elevated STK38L protein levels compared to cell lines that were not STK38L-dependent (Figure 1E). Collectively, these findings suggest that high STK38L mRNA and protein expression correlates well with STK38L dependency in PDAC cell lines. *STK38L* gene copy number gains can be found in a subset of cell lines, but do not correlate significantly with STK38L dependency.

### The kinase activity of STK38L is necessary to promote cell survival

**Figure 3:**
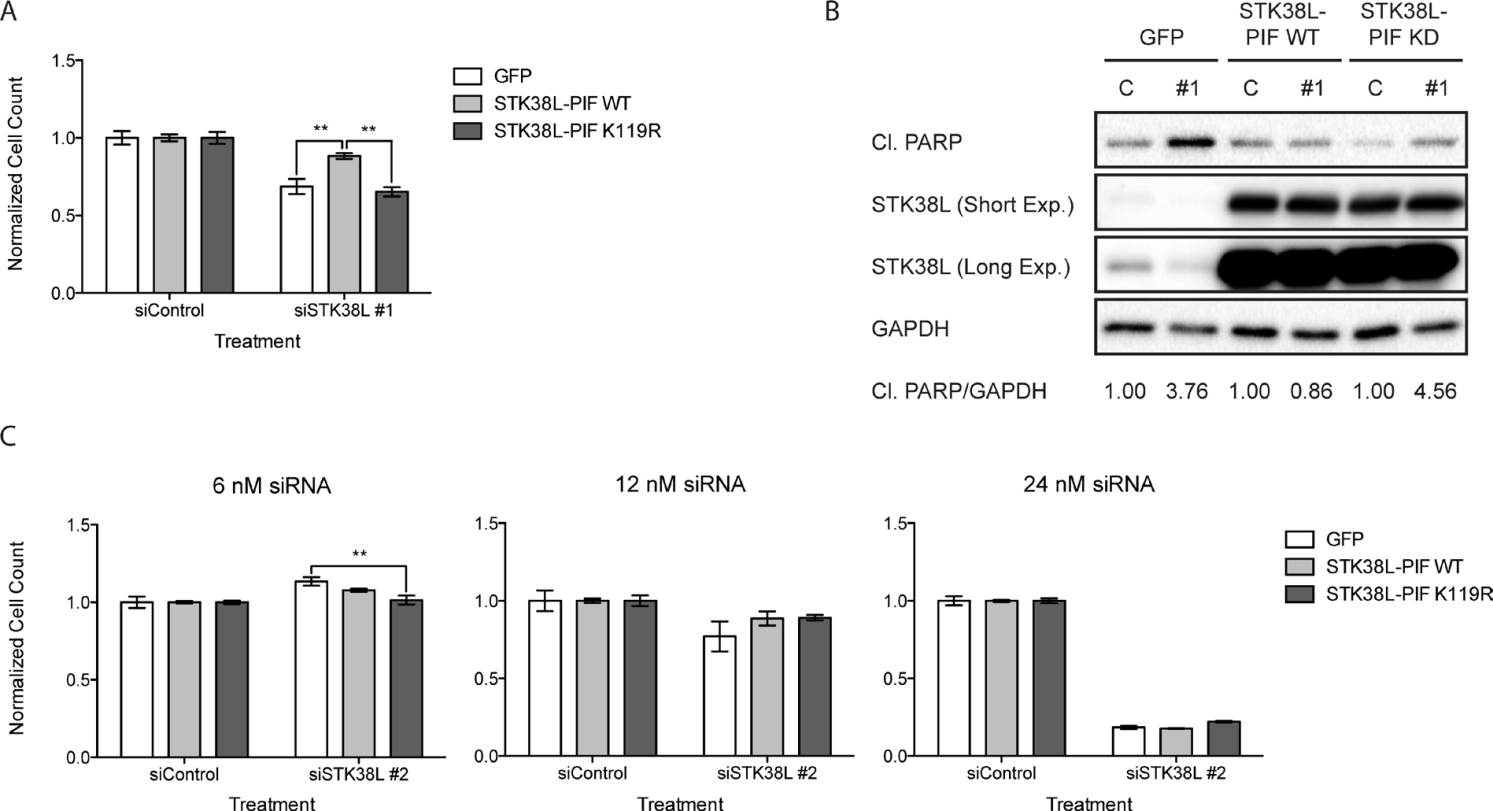
STK38L kinase activity is necessary to promote the survival of DAN-G cells (**A**) Cell number quantitation of DAN-G cells stably expressing GFP, HA-STK38L-PIF WT, or HA-STK38L-PIF K119R 48 h post-transfection with a siRNA directed against the 3′-UTR of *STK38L* at 6 nM final concentration. Data are the mean of five technical replicates +/− SEM. Statistical significance was assessed by one-way ANOVA with Tukey’s test for post-hoc validation (**−p < 0.01). (**B**) Western blot analysis of cleaved PARP levels following siRNA-mediated depletion of STK38L in DAN-G cells stably expressing GFP, HA-STK38L-PIF WT, or HA-STK38L-PIF K119R. GAPDH serves as a gel loading control. Protein band (cleaved PARP to GAPDH ratio) densitometric quantification values are indicated at the bottom. (**C**) Quantitation of DAN-G cells stably expressing GFP, HA-STK38L-PIF WT, or HA-STK38L-PIF K119R 48 h post-transfection with a siRNA directed against the coding sequence of STK38L. Data are the mean of five technical replicates +/− SEM. Statistical significance was assessed by one-way ANOVA with Tukey’s test for post-hoc validation (**−p < 0.01).

To validate the on-target specificity of STK38L-directed siRNA oligonucleotides, we performed a series of rescue experiments using a constitutively-activated variant of STK38L (STK38L-PIF), which contains a PRK2 hydrophobic motif at the C-terminus [23, 24]. We established DAN-G cells stably expressing GFP, HA-STK38L-PIF WT, or HA-STK38L-PIF K119R (kinase-dead variant) (Supplementary Figure 4). We did not observe any gross morphological or phenotypic abnormalities in cells expressing these STK38L variants. HA-STK38L-PIF K119R may be expected to have a dominant negative growth inhibitory effect. However, we did not observe any proliferative effects in DAN-G cells expressing the STK38L K119R mutant. This could be due to low expression levels of the mutant protein. We depleted endogenous STK38L in the stable DAN-G cell lines by transfection with an siRNA directed against the 3′ UTR of *STK38L* mRNA (siSTK38L #1), which does not target the exogenous STK38L mRNA. We quantified the total number of cells remaining following STK38L depletion by automated DAPI-stained nuclei counting (Figure 3A). Cells expressing GFP or HA-STK38L-PIF K119R showed reduced cell numbers following STK38L depletion. In contrast, cells expressing HA-STK38L-PIF WT were more proliferative following STK38L depletion (Figure 3A). Under similar experimental conditions, we analyzed changes in cleaved PARP by Western blotting (Figure 3B). DAN-G cells expressing GFP or HA-STK38L-PIF K119R exhibited nearly a four-fold increase in cleaved PARP when compared to the control treatment. However, cells expressing HA-STK38L-PIF WT exhibited no significant change in PARP cleavage. As an additional control, we used a siRNA directed against the coding sequence of *STK38L* (siSTK38L #2), which targets both endogenous and exogenous STK38L. This siRNA had similar antiproliferative effects in cell lines stably expressing GFP, HA-STK38L-PIF WT and HA-STK38L-PIF K119R (Figure 3C). Taken together, these findings demonstrate that the effects of STK38L-directed siRNAs on cell viability are on-target and that the kinase activity of STK38L is necessary for cell survival in DAN-G cells.

### STK38L depletion causes reduced clonogenic growth

**Figure 4:**
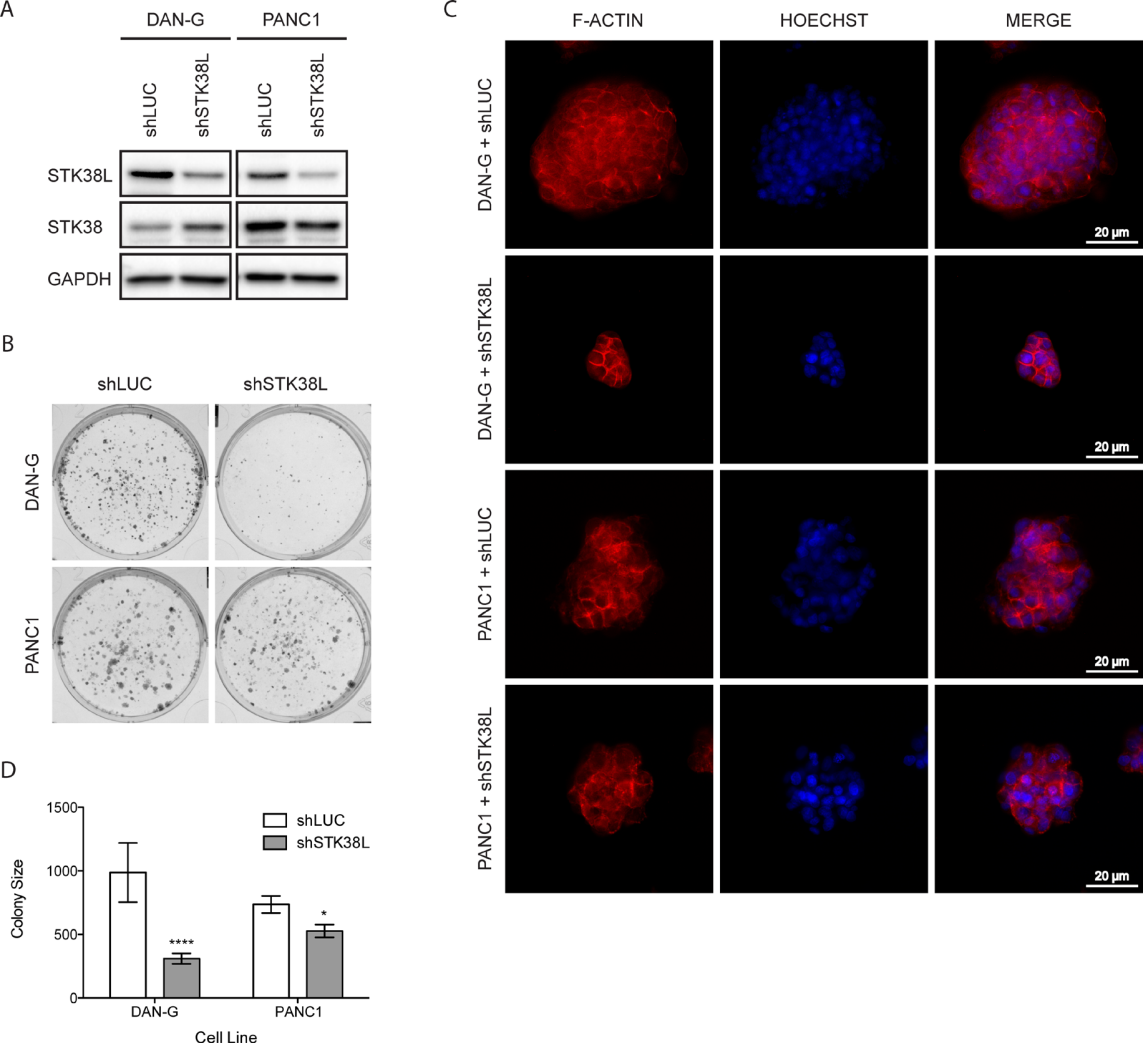
STK38L promotes clonogenic growth of DAN-G cells (**A**) Western blot analysis of stable DAN-G and PANC1 cell lines expressing shRNAs for luciferase (shLUC) or *STK38L* (shSTK38L). GAPDH serves as a gel loading control. (**B**) Representative images of colonies formed by DAN-G and PANC1 cells in 2D culture 11 days post-transduction with lentiviral shRNA expression vectors directed against luciferase or STK38L. (**C**) Representative images of colonies formed by DAN-G and PANC1 cells in Matrigel™ 7 days post-transduction with lentiviral shRNA expression vectors directed against luciferase or *STK38L*. Alexa594-Phalloidin-stained F-actin is visualized in red and DAPI-stained nuclear DNA in blue. Scale bar = 20 µm. Data are representative of two independent experiments. (**D**) Average colony size of DAN-G and PANC1 cells grown in Matrigel™ and imaged 7-days post-transduction with lentiviral shRNA expression vectors directed against luciferase or *STK38L*. Statistical significance was assessed by Student’s *t*-test (*−p < 0.05; ****−p < 0.0001).

To further validate the role of STK38L in promoting cell proliferation and survival, we performed a series of clonogenic assays, initially using 2D monolayer cell cultures. First, we stably depleted STK38L in DAN-G and PANC1 cells by lentiviral shRNA transduction and verified selective STK38L protein knockdown (Figure 4A). Then, we performed a clonogenic assay using the same STK38L-selective shRNA. STK38L-depleted DAN-G cells formed significantly fewer and smaller colonies compared to shLuciferase (shLUC)-expressing cells (Figure 4B). STK38L-depleted PANC1 cells also formed smaller colonies compared to control shLUC-expressing cells. However, the effects of STK38L depletion were relatively modest in PANC1 cells compared to DAN-G cells. Next, we analyzed clonogenic growth of DAN-G cells in 3D Matrigel™ assays, which provide a more physiologically relevant microenvironment that incorporates tumor cell-extracellular matrix (ECM) interactions. When grown in Matrigel™, STK38L-depleted DAN-G cells formed smaller colonies compared to shLuciferase (shLUC) expressing cells, as demonstrated by colony nuclear DNA and actin staining (Figure 4C). STK38L-depleted PANC1 cells also formed smaller colonies compared to control shLUC expressing cells. However, the effect of STK38L depletion was relatively modest in PANC1 cells when compared to DAN-G cells. Automated computer-assisted quantitation of colony size validated these results for shLUC and shSTK38L expressing DAN-G and PANC1 cells (Figure 4D). STK38L depletion caused a 68% reduction in the size of colonies formed by DAN-G cells but only a 28% reduction in the size of colonies formed by PANC1 cells. In summary, STK38L depletion causes substantially reduced clonogenic growth in DAN-G cells but only has a modest effect in PANC1 cells.

### STK38L depletion causes induction of LATS2 expression concomitant with cell death

To elucidate mechanisms underlying STK38L dependency in PDAC cell lines, we characterized the effects of STK38L depletion on expression levels of key proteins in KRAS and general tumor-associated signaling pathways including p21, MYC, MAPK, PI3K, and autophagy (Supplementary Figure 5A). STK38L depletion caused increased ERK and ribosomal S6 protein phosphorylation in some cell lines, including DAN-G. We also noted that LC3-II levels were moderately increased in a few cell lines following STK38L depletion, indicating a possible block in autophagy flux. This is concordant with previous reports of NDR kinase regulation of autophagosome formation [25]. However, this effect of STK38L depletion on LC3-II levels was not generalizable across all PDAC cell lines tested and did not correlate with PARP cleavage-associated cell death. In over half of the cell lines tested, particularly STK38L-dependent cell lines, STK38L depletion led to increased protein levels of p21, consistent with previous reports [20, 26, 27]. The context-dependent role of p21 in promoting or blocking apoptosis is unclear [28, 29]. Therefore, we tested a role for p21 in STK38L-regulated cell survival. To that end, we concomitantly depleted p21 and STK38L in DAN-G cells using combinations of siRNA oligonucleotides (Supplementary Figure 5B). Depletion of p21 alone had no effect on apoptotic cell death induction, as assessed by PARP cleavage. On the contrary, concomitant depletion of STK38L and p21 caused increased PARP cleavage compared to STK38L depletion alone. We also depleted p21 prior to STK38L depletion and, again, observed no significant effect on cell proliferation (Supplementary Figure 5C). Therefore, we conclude that increased p21 protein levels do not contribute to cytotoxicity following STK38L depletion.

**Figure 5:**
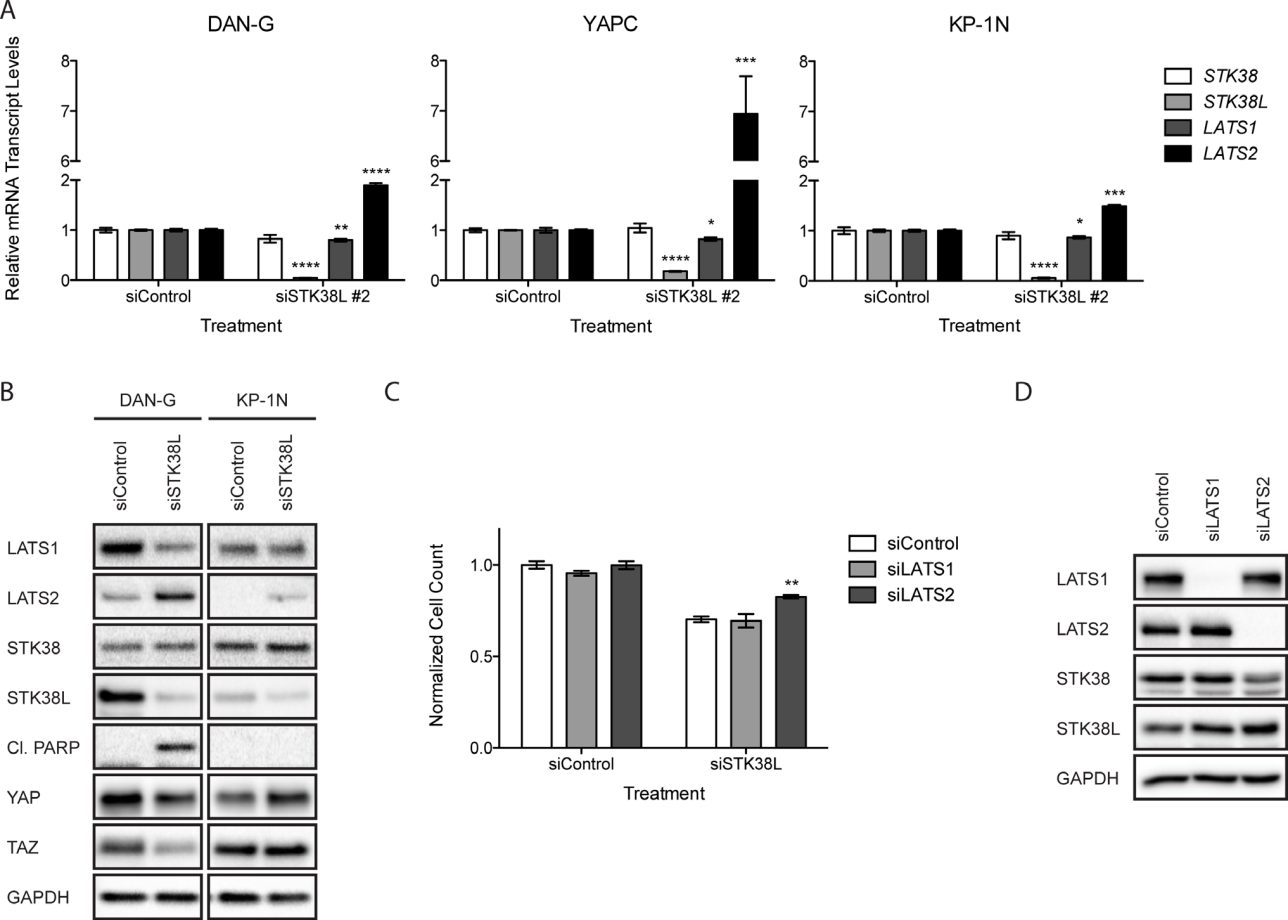
Increased LATS2 expression following STK38L depletion contributes to cytotoxicity (**A**) RT-qPCR analysis of relative mRNA transcript levels in DAN-G, YAPC, and KP-1N cells 24 h post-transfection with a single siRNA directed against *STK38L* (siSTK38L #2). Transcript levels are normalized to the reference gene *GAPDH*. Data are the mean of three technical replicates +/− SEM. Statistical significance was assessed by Student’s *t*-test (* -*p* < 0.05; **−p < 0.01; ***−p < 0.001; ****−p < 0.0001). (**B**) Western blot analysis of DAN-G and KP-1N cells 48 h post-transfection with siSTK38L #2. GAPDH serves as a gel loading control (**C**) Quantitation of DAN-G cell numbers 48 h post-transfection with siRNAs directed against *LATS1* or *LATS2* plus control siRNA or in combination with siSTK38L. Data are the mean of fifteen technical replicates +/− SEM and are representative of two independent experiments. Statistical significance was assessed by one-way ANOVA with Dunnett’s test for post-hoc validation (**−p < 0.01). (**D**) Western blot analysis of DAN-G cells 48 h post-transfection with pooled siRNAs directed against *LATS1* and *LATS2*. GAPDH serves as a gel loading control.

To further validate the on-target effects of siRNA-mediated STK38L depletion, we determined mRNA expression levels of related *STK38, LATS1* and *LATS2* kinase genes. STK38L depletion caused increased mRNA expression of *LATS2* but had no effects on *STK38* or *LATS1* mRNA transcript levels (Figure 5A). These effects of STK38L depletion on *LATS1/2* and *STK38/STK38L* mRNA expression were consistent with changes in protein expression levels as assessed by Western blotting (Figure 5B). Depletion of STK38L caused reduced expression of LATS1 in DAN-G cells but had no effect in KP-1N cells. In contrast, STK38L depletion caused increased protein levels of LATS2 in both cell lines. The effect of STK38L depletion on LATS2 mRNA and protein expression was more robust in DAN-G cells compared to KP-1N cells. Reciprocal control of LATS1/2 and STK38/STK38L expression levels has been documented in previous reports [30–32]. However, our studies represent the first report of STK38L-mediated control of LATS2 expression. Upon activation of the Hippo signaling pathway, LATS2 controls the phosphorylation, stability, and subcellular localization of the transcriptional co-activators YAP and TAZ [33, 34]. Increased LATS2 protein expression in DAN-G cells, following STK38L depletion, was associated with decreased levels of YAP and TAZ (Figure 5B). In contrast, STK38L depletion did not cause decreased YAP and TAZ protein expression in KP-1N cells.

Increased LATS2 protein expression can promote apoptosis in some contexts [35–37]. Thus, we hypothesized that induction of LATS2 expression contributes to cytotoxicity following STK38L depletion. To test this, we depleted STK38L alone or in combination with either LATS1 or LATS2 depletion in DAN-G cells. Depletion of either LATS1 or LATS2 alone had no significant effect on cell proliferation and viability (Figure 5C). STK38L depletion alone caused reduced cell proliferation and viability in DAN-G cells, as seen previously. This effect did not change significantly with co-depletion of LATS1. In contrast, co-depletion of LATS2 with STK38L caused a 13% increase in cell proliferation (Figure 5C). We confirmed on-target effects of siRNA co-transfections on LATS1/2 and STK38/STK38L protein expression levels by Western blotting (Figure 5D). Taken together, we conclude that the cytotoxicity associated with STK38L depletion in DAN-G cells occurs, in part, via induction of LATS2 expression.

**Figure 6:**
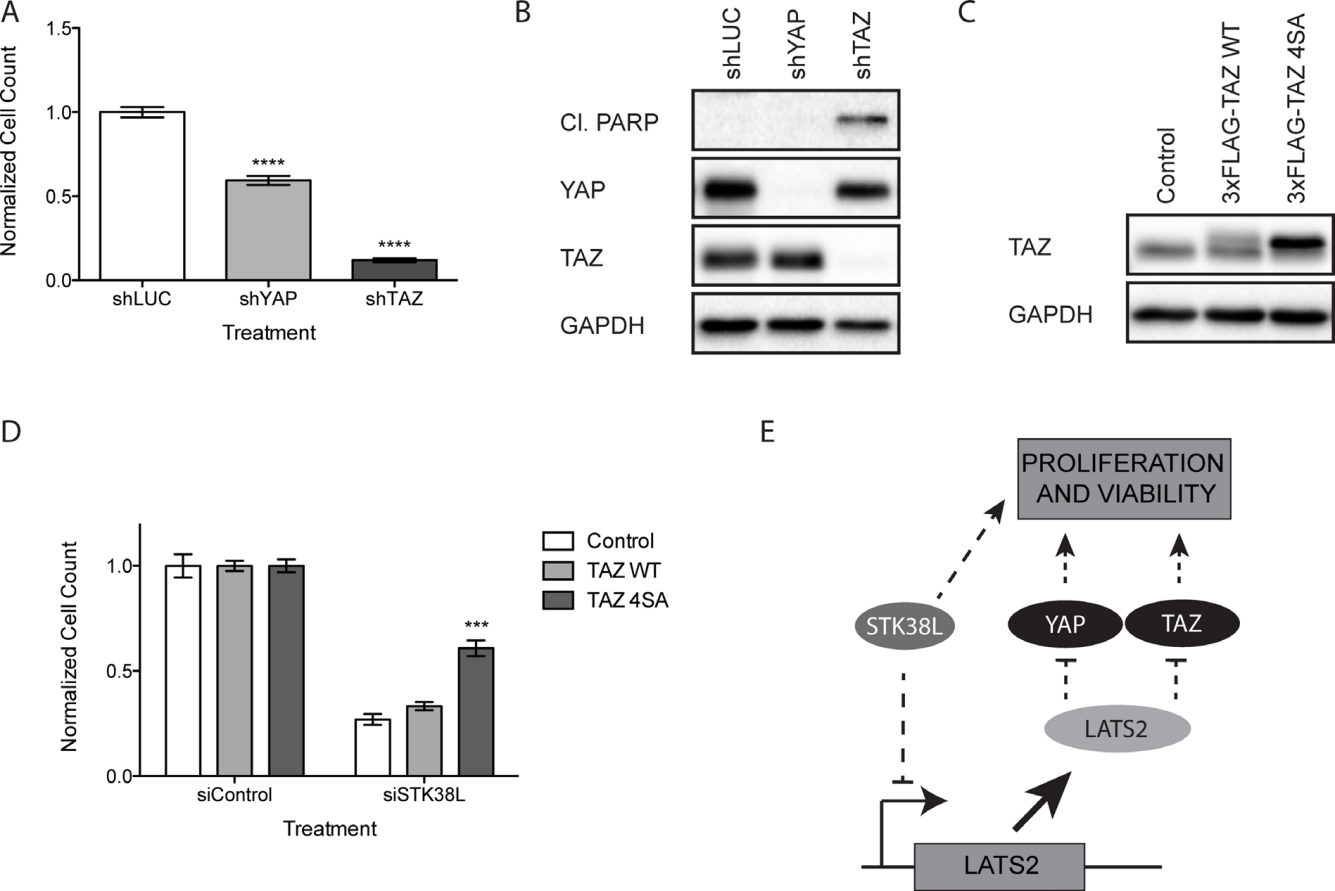
Loss of TAZ induces apoptosis in DAN-G cells (**A**) Quantitation of DAN-G cell numbers 72 h post-transduction with shRNAs directed against luciferase (shLUC), YAP (shYAP) or TAZ (shTAZ). Data are the mean of twelve technical replicates +/− SEM and are representative of two independent experiments. Statistical significance was assessed by one-way ANOVA with Dunnett’s test for post-hoc validation (****−p < 0.0001). (**B**) Western blot analysis of DAN-G cells 72 h post-transduction with lentiviral shRNA expression vectors directed against luciferase, YAP, or TAZ. GAPDH serves as a loading control. (**C**) Western blot analysis of DAN-G cells stably expressing an empty vector Control, 3xFLAG-TAZ WT, or 3xFLAG-TAZ 4SA. Both endogenous (lower band) and 3xFLAG-tagged exogenous (upper band) TAZ are shown. GAPDH serves as a gel loading control. (**D**) Quantitation of DAN-G cells stably expressing an empty vector Control, 3xFLAG-TAZ WT, or 3xFLAG-TAZ 4SA 48 h post-transfection with a siRNAs directed against STK38L at 25 nM final concentration. Data are the mean of eighteen technical replicates +/− SEM and are representative of two independent experiments. Statistical significance was assessed by one-way ANOVA with Dunnett’s test for post-hoc validation (***−p < 0.001). (**E**) Schematic representation of STK38L-mediated control of cellular proliferation and viability via suppression of LATS2 expression and concomitant activation of parallel survival pathways.

YAP and TAZ interact with the TEAD family of transcription factors to drive the expression of genes that promote cell survival and proliferation [38]. Thus, we hypothesized that reduced YAP/TAZ expression could confer antiproliferative effects in DAN-G cells, which are sensitive to STK38L depletion. To test this hypothesis, we depleted YAP/TAZ by lentiviral shRNA transduction in DAN-G cells. We then quantitated the total number of remaining cells by automated DAPI-stained nuclei counting (Figure 6A). Reductions in total cell number were observed following depletion of YAP or TAZ relative to the luciferase control. However, this effect was more robust following TAZ depletion. To determine whether the reductions in total cell number were due to apoptotic cell death, we analyzed changes in cleaved PARP under the same experimental conditions (Figure 6B). Cleaved PARP was observed in TAZ-depleted cells but not YAP-depleted cells. We conclude that loss of TAZ induces apoptosis in DAN-G cells whereas loss of YAP causes a reduction in cell proliferation.

To determine whether the loss of viability in DAN-G cells observed following STK38L depletion was due to a decrease in TAZ protein levels, we performed a rescue experiment using a mutant form of TAZ (4SA: S66A, S89A, S117A, and S311A) that cannot be phosphorylated by LATS2 [39]. We established DAN-G cells stably expressing an empty vector Control, 3xFLAG epitope-tagged TAZ WT (3xFLAG-TAZ WT), or 3xFLAG-TAZ 4SA (Figure 6C). Exogenous levels of TAZ WT were notably lower than TAZ 4SA, likely due to the increased protein stability of the 4SA mutant. We depleted STK38L in stable DAN-G cell lines and quantified the total number of cells remaining by automated DAPI-stained nuclei counting (Figure 6D). Cells expressing the empty vector Control or TAZ WT showed significantly reduced cell numbers following STK38L depletion (73% and 67% respectively). Comparatively, cells expressing TAZ 4SA showed a significantly weaker reduction in cell number following STK38L depletion (39%). Collectively, these findings suggest the cytotoxic effect associated with STK38L depletion is due, in part, to LATS2-mediated degradation of TAZ (Figure 6E).

### High STK38L mRNA expression is associated poor prognosis in PDAC patients

**Figure 7:**
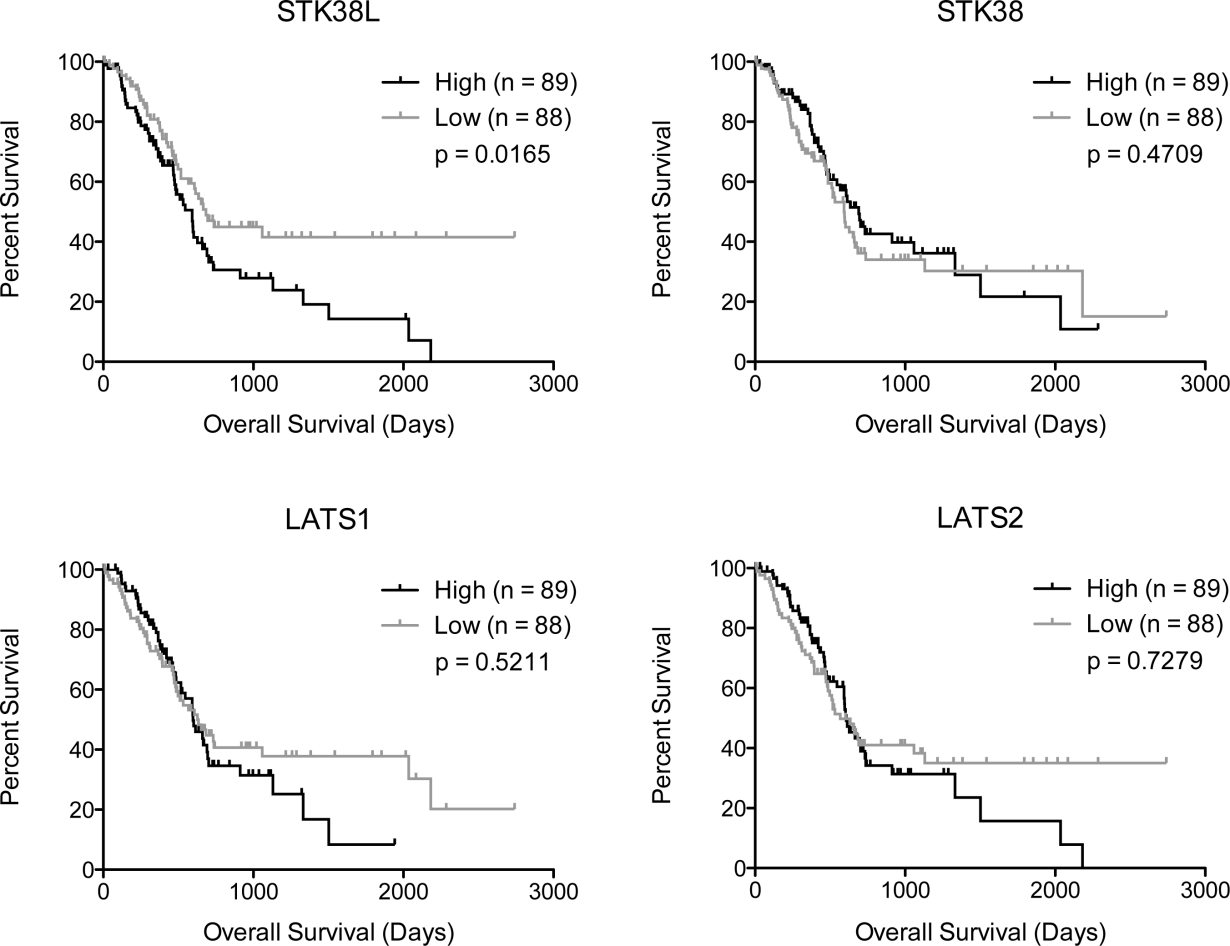
High STK38L expression is associated with decreased overall survival in PDAC patients Kaplan-Meier analysis of PDAC patient survival data showing the effects of indicated gene expression on overall survival. Statistical significance was assessed by Mantel–Cox test (log-rank *p*-value).

To determine whether *STK38L* expression correlates with overall survival of PDAC patients, we performed Kaplan-Meier analyses using publically-available RNA-seq-derived gene expression data from The Cancer Genome Atlas (TCGA-PAAD dataset) (Figure 7) [40]. We found that high *STK38L* mRNA expression associates with a significantly lower overall patient survival when compared to low *STK38L* expression (hazard ratio: 1.652, 95% confidence interval: 1.096–2.490, p = 0.0165). Of note, expression of *LATS1/2* and *STK38* kinase genes did not correlate with survival. As *STK38L* and *KRAS* are co-amplified in a subset of PDACs, we examined whether there is a relationship between *KRAS* expression and survival. We found that high *KRAS* expression was indeed associated with poor survival (hazard ratio: 1.766, 95% confidence interval: 1.174–2.656, p = 0.0063) (Supplementary Figure 6). To rule out the possibility that the lower overall survival associated with *STK38L* expression is a passenger effect, we analyzed the expression of other genes located in the chromosome 12p11–12p12 amplicon. Of the seven genes analyzed (*RASSF8*, *BHLHE41, SSPN, ITPR2, FGFR1OP2, TM7SF3* and *MED21)*, none associated significantly with patient outcome (*p* > 0.05) (Supplementary Figure 6). Therefore, we conclude that elevated *STK38L* and *KRAS* mRNA expression levels are specifically associated with poor patient outcome in PDAC cases.

## DISCUSSION

The inter- and intratumoral molecular heterogeneity of PDAC has confounded efforts to identify generally efficacious therapeutic agents. It is plausible that molecular subtype-selective targeted therapeutic agents could be effective in managing disease outcome by allowing for a more tailored and precise regimen. In this study, we identified *STK38L* gene amplification in a subset of human PDACs. Importantly, high *STK38L* gene expression associates with poor patient survival. Genetic *STK38L* depletion causes the selective killing of a subset of human PDAC cell lines, many of which have molecular hallmarks of the ADEX PDAC subtype [4]. Therefore, we implicate STK38L as a candidate, therapeutic target for a subset of ADEX-like PDACs. Analysis of a large PDAC cell line cohort demonstrated that *STK38L* gene copy number does not predict STK38L dependency. Our analysis of *STK38L* gene copy number was performed using a qPCR-based approach, which has inherent limitations. Future studies of *STK38L* amplification will utilize fluorescence *in situ* hybridization (FISH) to definitively quantitate *STK38L* gene copy number gain in cell lines, as well as primary tumors, and determine its utility as a biomarker. STK38L mRNA or protein expression levels may provide a more accurate biomarker for prediction of STK38L dependency in tumors. To further develop STK38L kinase inhibition as a therapeutic strategy, it will be necessary to identify additional biomarkers of STK38L dependency, such as co-occurring genetic alterations or gene expression signatures. Co-occurring, non-driver mutations may cooperate with oncogenic KRAS and STK38L to promote PDAC cell survival, lending complexity to the clinical potential of KRAS and STK38L-directed therapeutics. However, the identification of non-driver mutations and non-oncogene dependencies could allow for the development of potential combination-based therapies to effectively treat PDAC.

Previous studies demonstrate that STK38 and STK38L have redundant tumor suppressor function [18, 30]. Consistent with this finding, *STK38* and *STK38L* single gene deletions show no discernable phenotypes in mice, suggesting that selective STK38L inhibition will be well tolerated [32]. This provides support for STK38L as an attractive therapeutic target. Our findings highlight a context-dependent pro-tumorigenic role for STK38L. In concordance with our findings, a recent study demonstrates that STK38 promotes survival in some RAS-transformed cell lines by promoting clearance of damaged mitochondria [27]. However, STK38L does not compensate for loss of STK38 function in this context, indicating non-redundant function for these closely related kinases. Indeed, we found that STK38 expression levels do not affect cytotoxicity that is induced following STK38L depletion.

The pro-survival function of STK38L is closely related to KRAS oncogene dependency in PDAC cell lines. We observed highly significant correlations between STK38L and KRAS expression levels in PDAC cell lines as well as primary tumors. However, the mechanistic relationship between STK38L and KRAS remains unclear. STK38L depletion failed to induce consistent effects on downstream activation of KRAS effector pathways. Furthermore, expression of mutant KRAS 4A and 4B isoforms does not affect STK38L expression levels (Supplementary Figure 7). Therefore, we conclude that STK38L and KRAS function in parallel pathways to promote tumor cell survival. Further characterization of the mechanistic relationship between STK38L and KRAS will be critical to fully understand context-dependent tumor cell survival signaling networks in *KRAS* mutant PDACs.

Mechanistically, we demonstrate for the first time that STK38L depletion causes cell death, in part, via induction of LATS2 protein expression. LATS2 is a well-established tumor suppressor that, under normal physiological conditions, can restrict cell proliferation and promote apoptotic cell death. The mechanisms underlying LATS2-mediated cytotoxicity in PDAC cell lines have yet to be fully determined, but could involve perturbations in the phosphorylation and function of NDR/LATS substrates p21 and YAP [18, 20, 26, 37, 38]. YAP can be constitutively-activated in PDAC, and genetic ablation of YAP limits KRAS-driven PDAC progression in mouse models [41]. Consistent with this finding, we observed robust inhibition of cell proliferation following depletion of the transcriptional co-activator YAP or the closely related paralog TAZ. Further elucidation of the STK38L-LATS2-YAP/TAZ signaling axis will be interesting to explore in future studies. In summary, our findings highlight an important context-dependent role for STK38L kinase activity in promoting the survival of a distinct subset of KRAS-dependent PDAC cell lines via suppression of LATS2 apoptotic function. STK38L-dependent survival signaling represents an important vulnerability in subsets of PDAC cases that can potentially be exploited for therapeutic benefit. Thus, it will be important to develop ATP-competitive inhibitors to validate the kinase-dependent functions of STK38L in normal cells and in the pathophysiological context of STK38L-dependent tumor cell survival.

## MATERIALS AND METHODS

### Cell culture

Human-derived PDAC cell lines were cultured in Roswell Park Memorial Institute (RPMI) 1640 medium supplemented with 5% fetal bovine serum (FBS), 1mM sodium pyruvate, 100 Units/ml penicillin, and 100 µg/ml streptomycin. 293T cells were cultured in DMEM (Dulbecco’s Modified Eagle Medium) supplemented with 10% FBS. All cell lines were maintained in a humidified incubator at 37°C with 5% CO_2_.

### Analysis of human PDAC patient data

The Pancreatic Adenocarcinoma (PAAD) dataset provided by The Cancer Genome Atlas was analyzed using cBioPortal for Cancer Genomics maintained by the Memorial Sloan Kettering Cancer Center [42, 43]. This dataset was also analyzed using the UCSC Xena Functional Genomics Browser (http://genome.ucsc.edu/) to derive Kaplan-Meier plots of associations between selected gene expression levels and patient overall survival [44].

### Western blotting

Cells were washed in 1X PBS followed by lysis in 1X Laemlli Buffer supplemented with Halt Protease and Phosphatase Inhibitor Cocktail (ThermoFisher Inc.). Protein lysates were sonicated and normalized for total protein with a Bicinchoninic Acid (BCA) Assay Kit (Pierce). Proteins were resolved by SDS-PAGE and then transferred to a polyvinylidene fluoride (PVDF) membrane for Western blotting. Detection of proteins was carried out by enhanced chemiluminescence (ECL) using SuperSignal West-Pico or West-Dura (Thermo Fisher Scientific) reagents. Imaging was carried out with a Syngene G-Box XT4 system and the GeneSys imaging software. Detailed information regarding primary and secondary antibodies used for Western blotting is provided in Supplementary Table 2. The primary antibody for STK38L is previously described [45]. All antibodies were diluted in 2% w/v BSA in TBS-T.

### Cellular viability and quantitation assays

Cell viability assays were carried out in 96-well format with Alamar Blue reagent. Cells were subjected to esiRNA-mediated gene depletion for 72 h and subsequently incubated with 50 μg/ml alamarBlue for 1 h at 37°C/5% CO_2_. Alamar Blue fluorescence was measured with a FLUOstar OPTIMA Microplate Reader (BMG Labtech) at λ_Ex_/λ_Em_ 544/590 nm. The relative fraction of viable cells was determined by normalizing fluorescence intensity to parallel cultures transfected with control esiRNAs. Cell quantitation assays were carried out in 96-well format. Cells were subjected to siRNA-mediated gene depletion for 48 h or treatment with drug diluted in 0.1% DMSO for 24 h. Cells were fixed with 4% paraformaldehyde in 1X PBS and subsequently treated with 5 µg/ml Hoechst 33258 nuclear stain in 1X PBS for 20 min. Plates were scanned with a Cytation 3 Imaging Multi-Mode Reader (BioTek) at λ_Ex_/λ_Em_ 377/477 nm. Gen5 Microplate Reader and Imager Software was used to identify and count individual nuclei and determine total cell number. For image cytometry analyses of cellular subpopulations, DNA fluorescence intensity cut-off values were set in the Gen5 software to count cells in specific phases of the cell cycle, including sub-G1 (non-viable or apoptotic) cells.

### Recombinant lentivirus generation

Recombinant, replication-defective lentiviral particles were generated using a three-plasmid system previously described [46, 47]. Briefly, 293T cells were transfected with the desired lentiviral expression vector, the lentiviral packaging vector psPAX2 (gift from Didier Trono; Addgene plasmid #12260), and the lentiviral envelope vector pMD2.D-G VSV-G (gift from Didier Trono; Addgene plasmid #12259). Lentivirus production was induced the next day by treatment with DMEM supplemented with 30% FBS. The viral supernatant was collected 24 post-induction and syringe filtered to remove any potential cell contaminants. Target cells were subsequently spin-infected with lentiviral particles in the presence of 8µg/ml polybrene at 1200xg for 1 h. Selection of infected cells was carried out by treatment with 1 µg/ml puromycin.

### Clonogenic assays

For two-dimensional clonogenic assays, PDAC cells transduced with shRNA expression plasmids were seeded on a 6-well plate at a density of 1000 cells/well and maintained for 10 days. Colonies were fixed with 4% paraformaldehyde in 1X PBS and subsequently stained overnight with Geimsa dye (Ricca Chemical Company) diluted 1:50 in 1X PBS. Images of each well were captured with a Bio-Rad ChemiDoc XRS+ using the Image Lab™ Software. Three-dimensional clonogenic assays were performed using a method previously described [48]. PDAC cell lines transduced with shRNA expression plasmids were seeded on an 8-well chamber slide (Lab-Tek) coated with 200 µl Matrigel™ (Corning) diluted 1:1 in serum-free DMEM and maintained for 6 days. Representative images of each well were captured by bright-field microscopy and subsequently analyzed using CellProfiler software to determine average colony size [49]. For immunofluorescence imaging, colonies were fixed with 4% paraformaldehyde in 1X PBS and permeabilized with 0.1% Triton X-100. Colonies were sequentially incubated with 132 nM Alexa594-conjugated phalloidin in 1× PBS to stain actin filaments (Thermo Fisher Scientific) and then 2 µg/ml Hoechst 33256 dye in 1X PBS to stain nuclear DNA. Image acquisition was performed with a Cytation 3 Imaging Multi-Mode Reader (BioTek) at 10× magnification.

### Quantitative PCR-based gene expression analyses

RNA was isolated from cells with an RNeasy Mini Kit (Qiagen Inc.) and subjected to reverse transcription using a High Capacity Reverse Transcription Kit (Applied Biosystems Inc.). Diluted cDNA was used in TaqMan or SYBR Green-based qPCR assays. Relative quantitation determinations were obtained with a StepOne Real-Time PCR System (Applied Biosystems). Primer sequences used for SYBR Green-based assays are listed in Supplementary Table 3. Relative mRNA transcript levels were normalized to the reference gene *GAPDH* to allow for comparisons between cell lines.

### Gene copy number assay

Genomic DNA was isolated from cells with a QIAamp DNA Mini Kit (Qiagen Inc). *STK38L* gene copy number was assessed by genomic qPCR using a TaqMan Copy Number Assay Kit (Applied Biosystems). Recommended/validated probes for *STK38L* and the reference gene *RPPH1*/RNaseP were used in this assay. Relative quantitation determinations were obtained with a StepOne Real-Time PCR System (Applied Biosystems). *STK38L* gene copy number was normalized to the reference gene *RPPH1*/RNaseP to allow for comparisons between cell lines.

### Derivation of STK38L dependency

STK38L dependency was determined using a method similar to the one previously described for KRAS dependency [6]. The relative fraction of surviving cells following esiRNA-mediated depletion of STK38L was determined by Alamar Blue assay. The inverse of this fraction was then calculated to provide a quantitative value for STK38L dependency.

### siRNA transfections

Cells were transfected with siRNAs in 96-, 12-, or 6-well format. Transfections were performed in antibiotic-free media using a ratio of 20 pmol siRNA/µl Lipofectamine RNAiMax Transfection Reagent (Thermo Fisher Scientific).

### Immunohistochemistry (IHC)

STK38L expression was assessed in human PDAC samples by immunohistochemistry. Paraffin-embedded tumor microarrays (TMAs) were developed using samples that were obtained from consenting patients under IRB approved protocols (Massachusetts General Hospital/Dana Farber/Harvard Cancer Center, Department of Pathology). TMAs were stained using a Leica Bond III auto-stainer (Leica Biosystems) according to the manufacturer’s protocol. Antigen retrieval was carried out with Sodium Citrate (pH 6.0), and a rabbit polyclonal antibody directed against STK38L was applied to the TMAs at a dilution of 1:250. Tissue sections were counter-stained with hematoxylin. Slides were imaged using an Aperio ScanScope CS system (Leica Biosystems).

### Cell Lines, reagents, plasmid constructs

Cell lines were obtained from commercial sources and have been characterized previously (Supplementary Table 1) [6, 50–59]. Anisomycin was purchased from Sigma Aldrich Inc. Endoribonuclease-prepared siRNAs (esiRNAs) directed against *STK38L* were purchased from Sigma-Aldrich. Dicer substrate siRNAs (dsRNAs) directed against *STK38L* were purchased from Integrated DNA Technologies (IDT) Inc. SMARTpool siRNAs directed against *LATS1*, *LATS2*, and *CDKN1A*/p21 were purchased from Dharmacon Inc. Target sequences for all siRNAs are listed in Supplementary Table 4. For rescue experiments, HA-STK38L-PIF WT and HA-STK38L-PIF K119R were PCR-amplified from pcDNA3 and cloned into the pDONR223 Gateway donor vector. Primer sequences are listed in Supplementary Table 5. Genes were subsequently transferred to pLEX307 lentiviral-based expression plasmid (gift from David Root; Addgene plasmid #41392) by Gateway cloning (Invitrogen). Lentiviral pLKO.1 shRNA expression vectors for luciferase and STK38L were obtained from the RNAi Consortium (TRC – Broad Institute of Harvard/MIT). Lentiviral pLKO.1 shRNA expression vectors for YAP and TAZ have been described previously [60, 61]. The target sequences for all shRNAs are listed in Supplementary Table 6. 3xFLAG-TAZ WT/4SA expression constructs are in the pLVX-Tight-puro vector backbone and have been described previously [61]. KRAS 4A/4B expression constructs are in the pLenti-pGK vector backbone and have been described previously [12].

## SUPPLEMENTARY MATERIALS FIGURES AND TABLES



## References

[1] Siegel RL, Miller KD, Jemal A (2016). Cancer statistics, 2016. CA Cancer J Clin.

[2] Burris HA, Moore MJ, Andersen J, Green MR, Rothenberg ML, Modiano MR, Cripps MC, Portenoy RK, Storniolo AM, Tarassoff P, Nelson R, Dorr FA (1997). Improvements in survival and clinical benefit with gemcitabine as first-line therapy for patients with advanced pancreas cancer: a randomized trial. J Clin Oncol.

[3] Jones S, Zhang X, Parsons DW, Lin JC, Leary RJ, Angenendt P, Mankoo P, Carter H, Kamiyama H, Jimeno A, Hong SM, Fu B, Lin MT (2008). Core signaling pathways in human pancreatic cancers revealed by global genomic analyses. Science.

[4] Bailey P, Chang DK, Nones K, Johns AL, Patch AM, Gingras MC, Miller DK, Christ AN, Bruxner TJ, Quinn MC, Nourse C, Murtaugh LC, Harliwong I (2016). Genomic analyses identify molecular subtypes of pancreatic cancer. Nature.

[5] Collisson EA, Sadanandam A, Olson P, Gibb WJ, Truitt M, Gu S, Cooc J, Weinkle J, Kim GE, Jakkula L, Feiler HS, Ko AH, Olshen AB (2011). Subtypes of pancreatic ductal adenocarcinoma and their differing responses to therapy. Nat Med.

[6] Singh A, Greninger P, Rhodes D, Koopman L, Violette S, Bardeesy N, Settleman J (2009). A gene expression signature associated with “K-Ras addiction” reveals regulators of EMT and tumor cell survival. Cancer Cell.

[7] Collins MA, Bednar F, Zhang Y, Brisset JC, Galban S, Galban CJ, Rakshit S, Flannagan KS, Adsay NV, Pasca di Magliano M (2012). Oncogenic Kras is required for both the initiation and maintenance of pancreatic cancer in mice. J Clin Invest.

[8] Downward J (2003). Targeting RAS signalling pathways in cancer therapy. Nat Rev Cancer.

[9] Cox AD, Fesik SW, Kimmelman AC, Luo J, Der CJ (2014). Drugging the undruggable RAS: Mission possible?. Nat Rev Drug Discov.

[10] Stephen AG, Esposito D, Bagni RK, McCormick F (2014). Dragging ras back in the ring. Cancer Cell.

[11] Luo J, Solimini NL, Elledge SJ (2009). Principles of cancer therapy: oncogene and non-oncogene addiction. Cell.

[12] Singh A, Sweeney MF, Yu M, Burger A, Greninger P, Benes C, Haber DA, Settleman J (2012). TAK1 inhibition promotes apoptosis in KRAS-dependent colon cancers. Cell.

[13] Heidenblad M, Lindgren D, Veltman JA, Jonson T, Mahlamaki EH, Gorunova L, van Kessel AG, Schoenmakers EF, Hoglund M (2005). Microarray analyses reveal strong influence of DNA copy number alterations on the transcriptional patterns in pancreatic cancer: implications for the interpretation of genomic amplifications. Oncogene.

[14] Rodriguez S, Jafer O, Goker H, Summersgill BM, Zafarana G, Gillis AJ, van Gurp RJ, Oosterhuis JW, Lu YJ, Huddart R, Cooper CS, Clark J, Looijenga LH (2003). Expression profile of genes from 12p in testicular germ cell tumors of adolescents and adults associated with i(12p) and amplification at 12p11.2-p12.1. Oncogene.

[15] Dehan E, Ben-Dor A, Liao W, Lipson D, Frimer H, Rienstein S, Simansky D, Krupsky M, Yaron P, Friedman E, Rechavi G, Perlman M, Aviram-Goldring A (2007). Chromosomal aberrations and gene expression profiles in non-small cell lung cancer. Lung Cancer.

[16] Valtorta E, Misale S, Sartore-Bianchi A, Nagtegaal ID, Paraf F, Lauricella C, Dimartino V, Hobor S, Jacobs B, Ercolani C, Lamba S, Scala E, Veronese S (2013). KRAS gene amplification in colorectal cancer and impact on response to EGFR-targeted therapy. Int J Cancer.

[17] Weinstein IB, Joe A (2008). Oncogene addiction. Cancer Res.

[18] Zhang L, Tang F, Terracciano L, Hynx D, Kohler R, Bichet S, Hess D, Cron P, Hemmings BA, Hergovich A, Schmitz-Rohmer D (2015). NDR functions as a physiological YAP1 kinase in the intestinal epithelium. Curr Biol.

[19] Cornils H, Kohler RS, Hergovich A, Hemmings BA (2011). Downstream of human NDR kinases: impacting on c-myc and p21 protein stability to control cell cycle progression. Cell Cycle.

[20] Cornils H, Kohler RS, Hergovich A, Hemmings BA (2011). Human NDR kinases control G(1)/S cell cycle transition by directly regulating p21 stability. Mol Cell Biol.

[21] Abe S, Nagai T, Masukawa M, Okumoto K, Homma Y, Fujiki Y, Mizuno K (2017). Localization of Protein Kinase NDR2 to Peroxisomes and Its Role in Ciliogenesis. J Biol Chem.

[22] Kittler R, Putz G, Pelletier L, Poser I, Heninger AK, Drechsel D, Fischer S, Konstantinova I, Habermann B, Grabner H, Yaspo ML, Himmelbauer H, Korn B (2004). An endoribonuclease-prepared siRNA screen in human cells identifies genes essential for cell division. Nature.

[23] Stegert MR, Tamaskovic R, Bichsel SJ, Hergovich A, Hemmings BA (2004). Regulation of NDR2 protein kinase by multi-site phosphorylation and the S100B calcium-binding protein. J Biol Chem.

[24] Cook D, Hoa LY, Gomez V, Gomez M, Hergovich A (2014). Constitutively active NDR1-PIF kinase functions independent of MST1 and hMOB1 signalling. Cell Signal.

[25] Joffre C, Dupont N, Hoa L, Gomez V, Pardo R, Goncalves-Pimentel C, Achard P, Bettoun A, Meunier B, Bauvy C, Cascone I, Codogno P, Fanto M (2015). The Pro-apoptotic STK38 Kinase Is a New Beclin1 Partner Positively Regulating Autophagy. Curr Biol.

[26] Du Z, Tong X, Ye X (2013). Cyclin D1 promotes cell cycle progression through enhancing NDR1/2 kinase activity independent of cyclin-dependent kinase 4. J Biol Chem.

[27] Bettoun A, Joffre C, Zago G, Surdez D, Vallerand D, Gundogdu R, Sharif AA, Gomez M, Cascone I, Meunier B, White MA, Codogno P, Parrini MC (2016). Mitochondrial clearance by the STK38 kinase supports oncogenic Ras-induced cell transformation. Oncotarget.

[28] Huo JX, Metz SA, Li GD (2004). p53-independent induction of p21(waf1/cip1) contributes to the activation of caspases in GTP-depletion-induced apoptosis of insulin-secreting cells. Cell Death Differ.

[29] Hernandez AM, Colvin ES, Chen YC, Geiss SL, Eller LE, Fueger PT (2013). Upregulation of p21 activates the intrinsic apoptotic pathway in beta-cells. Am J Physiol Endocrinol Metab.

[30] Cornils H, Stegert MR, Hergovich A, Hynx D, Schmitz D, Dirnhofer S, Hemmings BA (2010). Ablation of the kinase NDR1 predisposes mice to the development of T cell lymphoma. Sci Signal.

[31] Moroishi T, Park HW, Qin B, Chen Q, Meng Z, Plouffe SW, Taniguchi K, Yu FX, Karin M, Pan D, Guan KL (2015). A YAP/TAZ-induced feedback mechanism regulates Hippo pathway homeostasis. Genes Dev.

[32] Schmitz-Rohmer D, Probst S, Yang ZZ, Laurent F, Stadler MB, Zuniga A, Zeller R, Hynx D, Hemmings BA, Hergovich A (2015). NDR Kinases Are Essential for Somitogenesis and Cardiac Looping during Mouse Embryonic Development. PLoS One.

[33] Zhao B, Li L, Tumaneng K, Wang CY, Guan KL (2010). A coordinated phosphorylation by Lats and CK1 regulates YAP stability through SCF(beta-TRCP). Genes Dev.

[34] Liu CY, Zha ZY, Zhou X, Zhang H, Huang W, Zhao D, Li T, Chan SW, Lim CJ, Hong W, Zhao S, Xiong Y, Lei QY (2010). The hippo tumor pathway promotes TAZ degradation by phosphorylating a phosphodegron and recruiting the SCF{beta}-TrCP E3 ligase. J Biol Chem.

[35] Kamikubo Y, Takaori-Kondo A, Uchiyama T, Hori T (2003). Inhibition of cell growth by conditional expression of kpm, a human homologue of Drosophila warts/lats tumor suppressor. J Biol Chem.

[36] Aylon Y, Ofir-Rosenfeld Y, Yabuta N, Lapi E, Nojima H, Lu X, Oren M (2010). The Lats2 tumor suppressor augments p53-mediated apoptosis by promoting the nuclear proapoptotic function of ASPP1. Genes Dev.

[37] Suzuki H, Yabuta N, Okada N, Torigata K, Aylon Y, Oren M, Nojima H (2013). Lats2 phosphorylates p21/CDKN1A after UV irradiation and regulates apoptosis. J Cell Sci.

[38] Meng Z, Moroishi T, Guan KL (2016). Mechanisms of Hippo pathway regulation. Genes Dev.

[39] Lei QY, Zhang H, Zhao B, Zha ZY, Bai F, Pei XH, Zhao S, Xiong Y, Guan KL (2008). TAZ promotes cell proliferation and epithelial-mesenchymal transition and is inhibited by the hippo pathway. Mol Cell Biol.

[40] Hudson TJ, Anderson W, Artez A, Barker AD, Bell C, Bernabe RR, Bhan MK, Calvo F, Eerola I, Gerhard DS, Guttmacher A, Guyer M, International Cancer Genome C (2010). International network of cancer genome projects. Nature.

[41] Zhang W, Nandakumar N, Shi Y, Manzano M, Smith A, Graham G, Gupta S, Vietsch EE, Laughlin SZ, Wadhwa M, Chetram M, Joshi M, Wang F (2014). Downstream of mutant KRAS, the transcription regulator YAP is essential for neoplastic progression to pancreatic ductal adenocarcinoma. Sci Signal.

[42] Cerami E, Gao J, Dogrusoz U, Gross BE, Sumer SO, Aksoy BA, Jacobsen A, Byrne CJ, Heuer ML, Larsson E, Antipin Y, Reva B, Goldberg AP (2012). The cBio cancer genomics portal: an open platform for exploring multidimensional cancer genomics data. Cancer Discov.

[43] Gao J, Aksoy BA, Dogrusoz U, Dresdner G, Gross B, Sumer SO, Sun Y, Jacobsen A, Sinha R, Larsson E, Cerami E, Sander C, Schultz N (2013). Integrative analysis of complex cancer genomics and clinical profiles using the cBioPortal. Sci Signal.

[44] Kent WJ, Sugnet CW, Furey TS, Roskin KM, Pringle TH, Zahler AM, Haussler D (2002). The human genome browser at UCSC. Genome Res.

[45] Vichalkovski A, Gresko E, Cornils H, Hergovich A, Schmitz D, Hemmings BA (2008). NDR kinase is activated by RASSF1A/MST1 in response to Fas receptor stimulation and promotes apoptosis. Curr Biol.

[46] Naldini L, Blomer U, Gallay P, Ory D, Mulligan R, Gage FH, Verma IM, Trono D (1996). In *vivo* gene delivery and stable transduction of nondividing cells by a lentiviral vector. Science.

[47] Moffat J, Grueneberg DA, Yang X, Kim SY, Kloepfer AM, Hinkle G, Piqani B, Eisenhaure TM, Luo B, Grenier JK, Carpenter AE, Foo SY, Stewart SA (2006). A lentiviral RNAi library for human and mouse genes applied to an arrayed viral high-content screen. Cell.

[48] Lee GY, Kenny PA, Lee EH, Bissell MJ (2007). Three-dimensional culture models of normal and malignant breast epithelial cells. Nat Methods.

[49] Carpenter AE, Jones TR, Lamprecht MR, Clarke C, Kang IH, Friman O, Guertin DA, Chang JH, Lindquist RA, Moffat J, Golland P, Sabatini DM (2006). CellProfiler: image analysis software for identifying and quantifying cell phenotypes. Genome Biol.

[50] Barretina J, Caponigro G, Stransky N, Venkatesan K, Margolin AA, Kim S, Wilson CJ, Lehar J, Kryukov GV, Sonkin D, Reddy A, Liu M, Murray L (2012). The Cancer Cell Line Encyclopedia enables predictive modelling of anticancer drug sensitivity. Nature.

[51] Kaufman JM, Yamada T, Park K, Timmers CD, Amann JM, Carbone DP (2017). A Transcriptional Signature Identifies LKB1 Functional Status as a Novel Determinant of MEK Sensitivity in Lung Adenocarcinoma. Cancer Res.

[52] Aguirre AJ, Brennan C, Bailey G, Sinha R, Feng B, Leo C, Zhang Y, Zhang J, Gans JD, Bardeesy N, Cauwels C, Cordon-Cardo C, Redston MS (2004). High-resolution characterization of the pancreatic adenocarcinoma genome. Proc Natl Acad Sci U S A.

[53] Tzatsos A, Paskaleva P, Ferrari F, Deshpande V, Stoykova S, Contino G, Wong KK, Lan F, Trojer P, Park PJ, Bardeesy N (2013). KDM2B promotes pancreatic cancer via Polycomb-dependent and -independent transcriptional programs. J Clin Invest.

[54] Heidenblad M, Schoenmakers EF, Jonson T, Gorunova L, Veltman JA, van Kessel AG, Hoglund M (2004). Genome-wide array-based comparative genomic hybridization reveals multiple amplification targets and novel homozygous deletions in pancreatic carcinoma cell lines. Cancer Res.

[55] Moore PS, Sipos B, Orlandini S, Sorio C, Real FX, Lemoine NR, Gress T, Bassi C, Kloppel G, Kalthoff H, Ungefroren H, Lohr M, Scarpa A (2001). Genetic profile of 22 pancreatic carcinoma cell lines. Analysis of K-ras, p53, p16 and DPC4/Smad4. Virchows Arch.

[56] Hayes TK, Neel NF, Hu C, Gautam P, Chenard M, Long B, Aziz M, Kassner M, Bryant KL, Pierobon M, Marayati R, Kher S, George SD (2016). Long-Term ERK Inhibition in KRAS-Mutant Pancreatic Cancer Is Associated with MYC Degradation and Senescence-like Growth Suppression. Cancer Cell.

[57] Mazur PK, Herner A, Mello SS, Wirth M, Hausmann S, Sanchez-Rivera FJ, Lofgren SM, Kuschma T, Hahn SA, Vangala D, Trajkovic-Arsic M, Gupta A, Heid I (2015). Combined inhibition of BET family proteins and histone deacetylases as a potential epigenetics-based therapy for pancreatic ductal adenocarcinoma. Nat Med.

[58] Stojanovic N, Hassan Z, Wirth M, Wenzel P, Beyer M, Schafer C, Brand P, Kroemer A, Stauber RH, Schmid RM, Arlt A, Sellmer A, Mahboobi S (2017). HDAC1 and HDAC2 integrate the expression of p53 mutants in pancreatic cancer. Oncogene.

[59] Suzuki A, Shibata T, Shimada Y, Murakami Y, Horii A, Shiratori K, Hirohashi S, Inazawa J, Imoto I (2008). Identification of SMURF1 as a possible target for 7q21.3-22.1 amplification detected in a pancreatic cancer cell line by in-house array-based comparative genomic hybridization. Cancer Sci.

[60] Zhao B, Ye X, Yu J, Li L, Li W, Li S, Yu J, Lin JD, Wang CY, Chinnaiyan AM, Lai ZC, Guan KL (2008). TEAD mediates YAP-dependent gene induction and growth control. Genes Dev.

[61] Hiemer SE, Szymaniak AD, Varelas X (2014). The transcriptional regulators TAZ and YAP direct transforming growth factor beta-induced tumorigenic phenotypes in breast cancer cells. J Biol Chem.

