# Supplementary material for: STK38L kinase ablation promotes loss of cell viability in a subset of KRAS-dependent pancreatic cancer cell lines
Source: Oncotarget. 2017 Sep 11;8(45):78556–72. doi: 10.18632/oncotarget.20833 (PMC5667982; doi:10.18632/oncotarget.20833)
Supplement: Supplementary file 1 [file oncotarget-08-78556-s001.pdf]

STK38L kinase ablation promotes loss of cell viability in a subset of KRAS-dependent pancreatic cancer cell lines

SUPPLEMENTARY MATERIALS

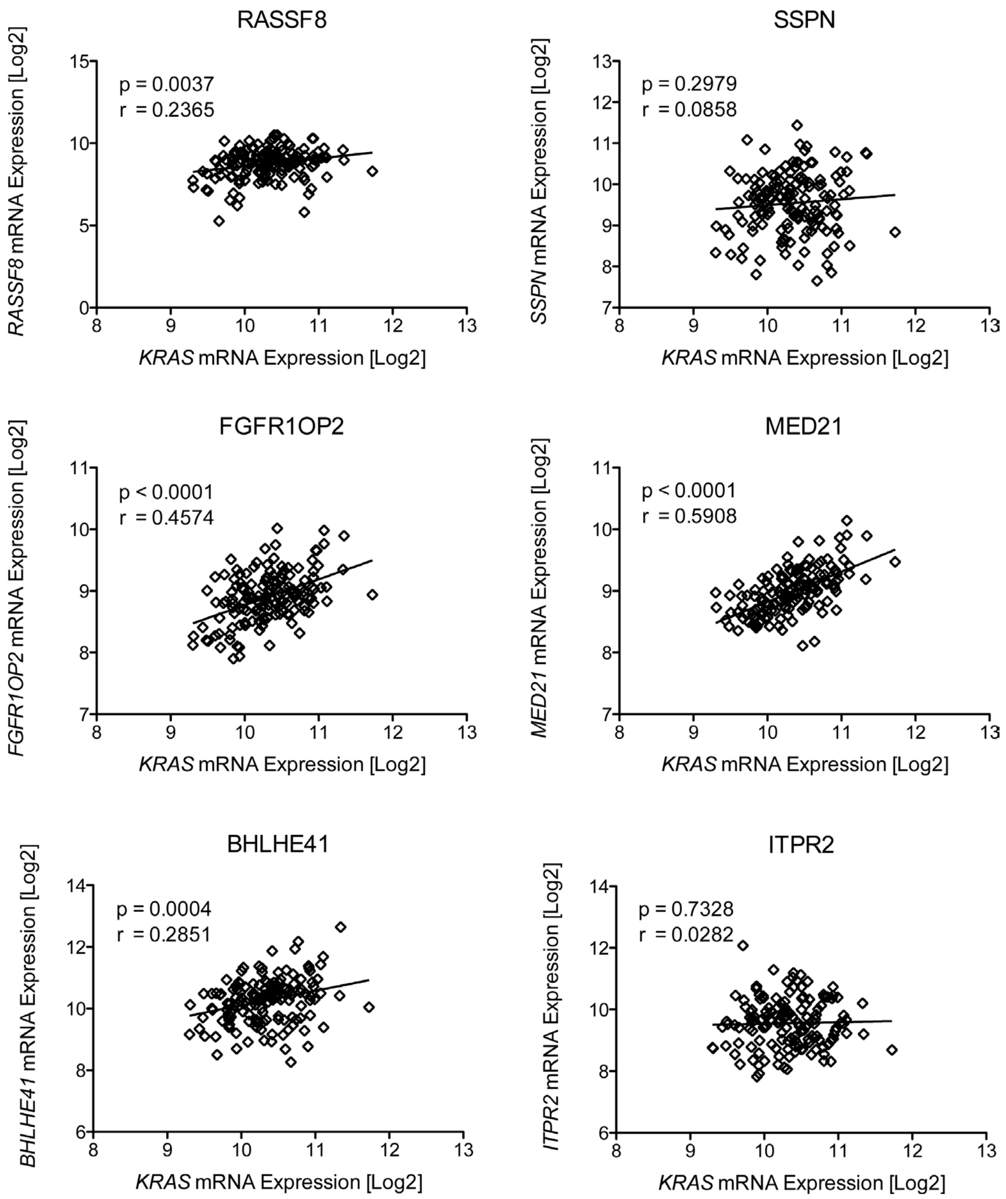

Supplementary Figure 1: Expression of *KRAS* does not always correlate with other co-amplified genes. Correlation analysis of 12p11-12p12 amplicon genes and *KRAS* mRNA expression (RNA-Seq V2 RSEM).

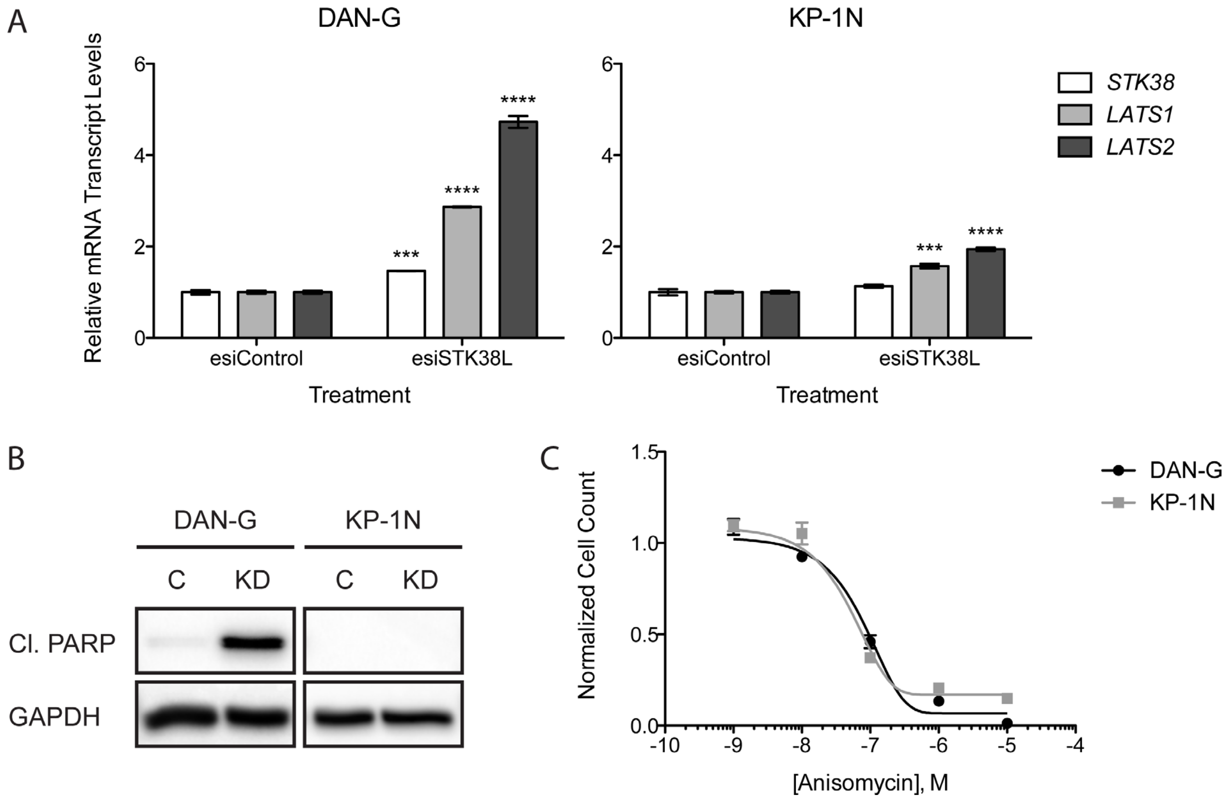

Supplementary Figure 2: KP-1N cells are insensitive to STK38L depletion. (**A**) RT-qPCR analysis of relative NDR/LATS kinase mRNA transcript levels for DAN-G and KP-1N cells 24 h post-transfection with pooled esiRNAs directed against *STK38L*. Transcript levels are normalized to the reference gene *GAPDH*. Data are the mean of three technical replicates +/− SEM. Statistical significance was assessed by Student's t-test (***−$p < 0.001$; ****−$p < 0.0001$). (**B**) Western blot analysis of cell death induction in DAN-G and KP-1N cells 48 h post-transfection with pooled esiRNAs directed against *STK38L*. GAPDH serves as a gel loading control. (**C**) Dose-response curve of DAN-G and KP-1N cells treated with anisomycin. Data are the mean of five technical replicates +/− SEM at the indicated drug concentrations.

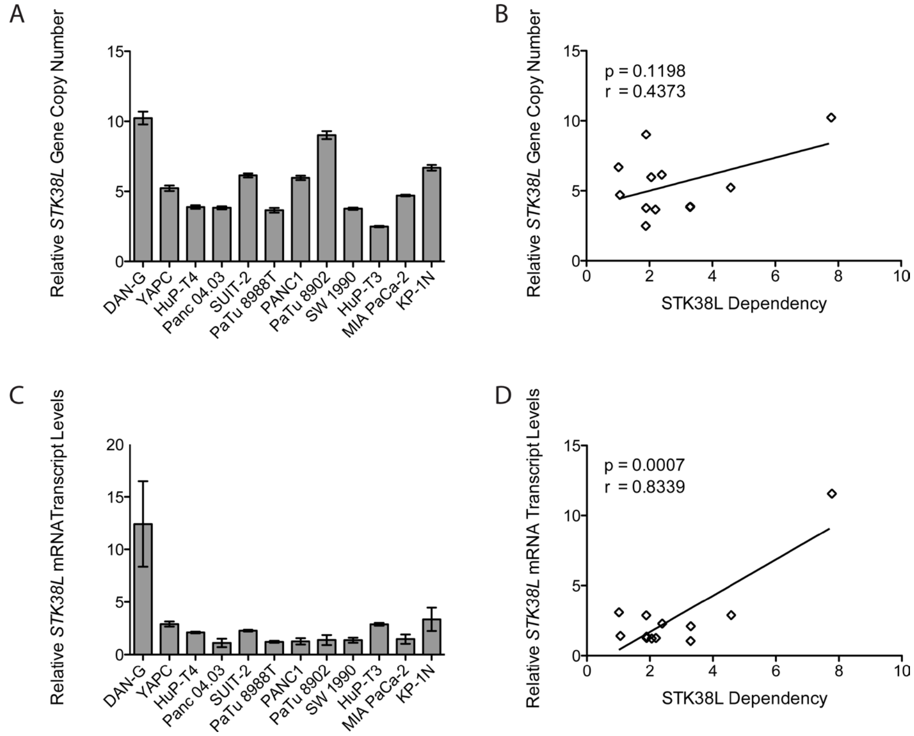

Supplementary Figure 3: Analysis of *STK38L* gene copy number and mRNA transcript levels in human PDAC cell lines. (**A**) Relative *STK38L* gene copy number in human PDAC cell lines as determined by genomic qPCR. Copy number values are normalized to the reference gene *RPPH1*/RNaseP. Data are the mean of four technical replicates +/− SEM. (**B**) Correlation analysis of *STK38L* dependency (calculated as the inverse of percent viability following esiSTK38L transfection) and gene copy number. (**C**) Relative *STK38L* mRNA transcript levels in human PDAC cell lines. Transcript levels were normalized to the reference gene *GAPDH*. Data are the mean of three technical replicates +/− SEM. (**D**) Correlation analysis of *STK38L* dependency and mRNA expression.

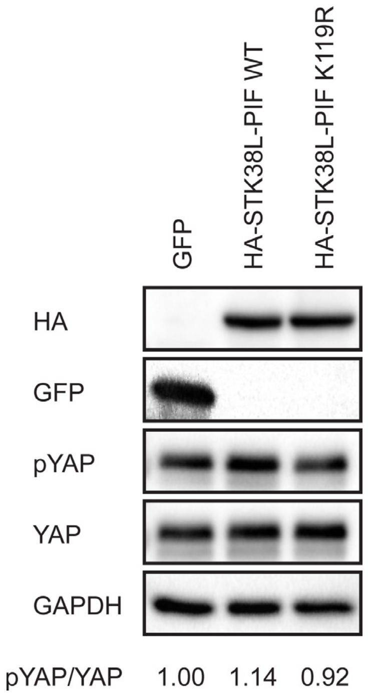

Supplementary Figure 4: Validation of DAN-G cells stably expressing STK38L-PIF WT and STK38L-PIF K119R. Western blot of DAN-G cells stably expressing GFP, HA-STK38L-PIF WT, or HA-STK38L-PIF K119R. GAPDH serves as a gel loading control.

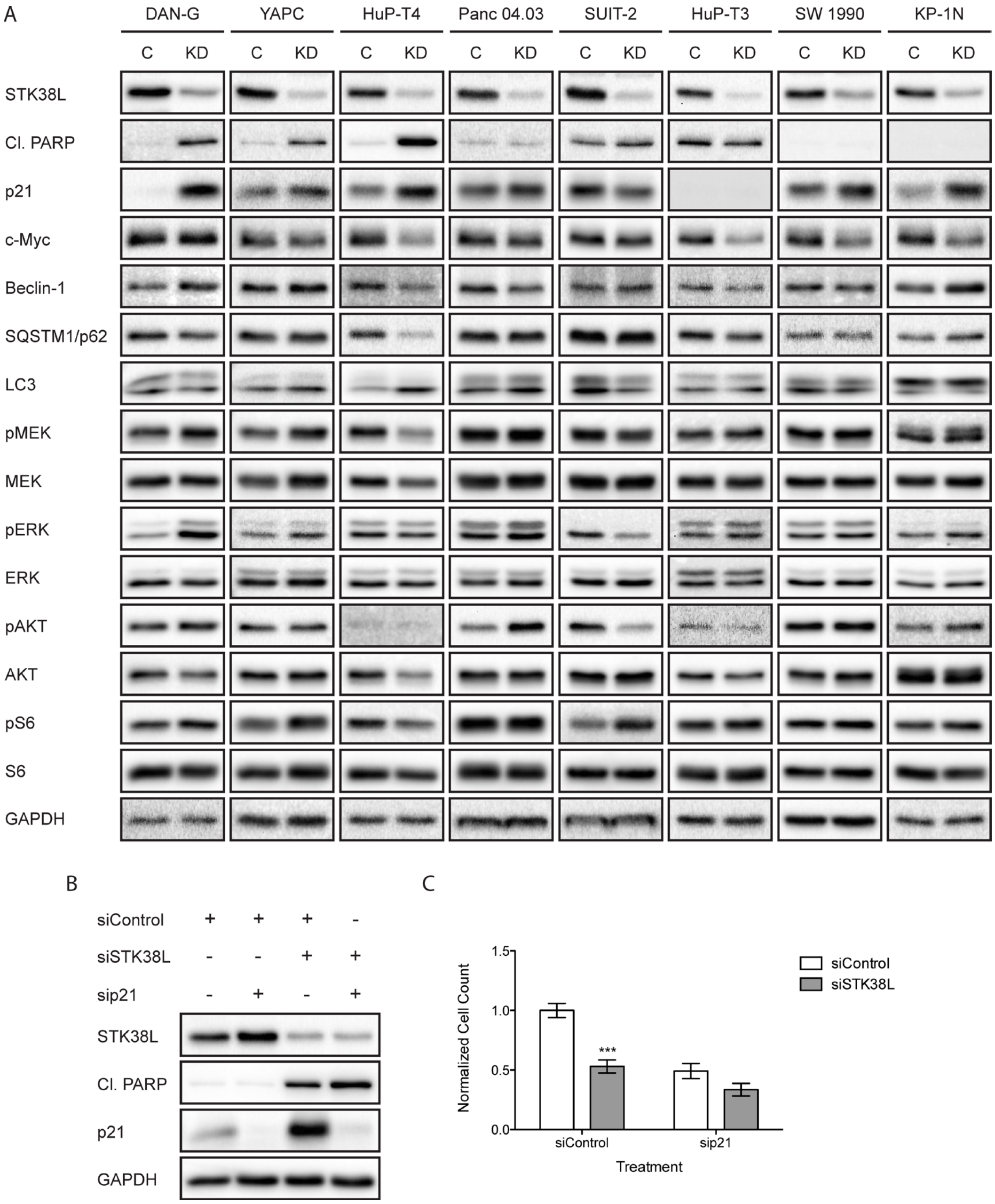

Supplementary Figure 5: STK38L depletion increases p21 protein abundance. (**A**) Western blot analysis of human PDAC cell lines 48 h post-transfection with two siRNAs directed against *STK38L* (siSTK38L #1 and #2) in combination at 10 nM final concentration. GAPDH is used as a gel loading control. Note: Different exposure times were used for individual panels. (**B**) Western blot analysis of DAN-G cells 48 h post-transfection with pooled siRNAs directed against *CDKN1A*/p21 (25 nM) and *STK38L* (10 nM). GAPDH serves as a gel loading control. (**C**) Quantitation of DAN-G cells 72 h post-transfection with siRNAs directed against *CDKN1A*/p21 and *STK38L* alone or in combination. Data are the mean of five technical replicates +/− SEM. Statistical significance was assessed by Student's t-test (***–$p < 0.001$).

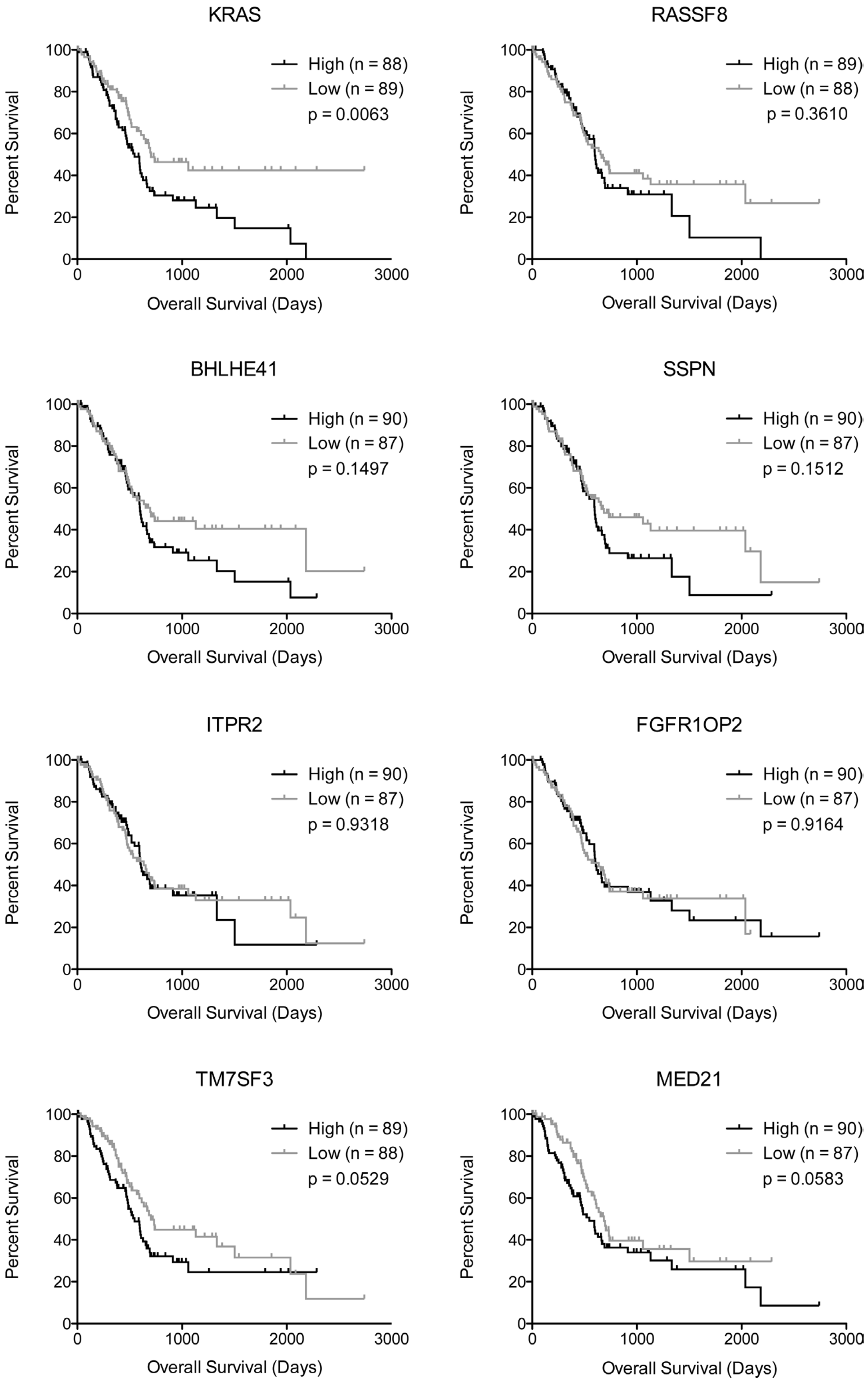

Supplementary Figure 6: High *KRAS* expression is associated with decrease overall survival in PDAC patients. Kaplan-Meier analysis of PDAC patient data showing the effect of *KRAS* expression, as well as the expression of other genes in the 12p11-12p12 chromosomal region, on overall survival. Statistical significance was assessed by Mantel–Cox test (log-rank *p*-value).

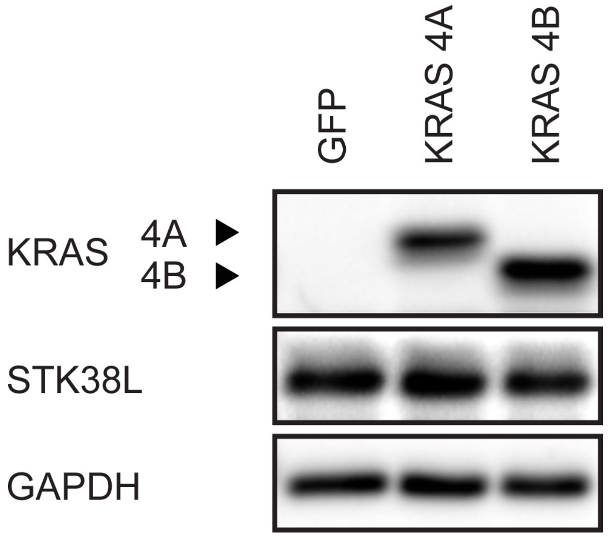

Supplementary Figure 7: KRAS does not modulate STK38L expression. Western blot of BxPC3 cells 72 h post-transduction with lentiviral expression vectors for GFP, KRAS 4A, or KRAS 4B. GAPDH serves as a gel loading control.

Supplementary Table 1: Cell Lines

	Gene Alterations				
**Cell Line**	***KRAS***	***CDKN2A***	***TP53***	***SMAD4***	**Source**
DAN-G	G12V	HD	933+16del.38	MUT	DSMZ
YAPC	G12V	HD	H179R	R515fs*22	DSMZ
HuP-T4	G12V	HD	1225T	N/A	DSMZ
Panc04.03	G12D	INS	G245S	N/A	ATCC
PaTu 8902	G12V	N/A	C176S	N/A	DSMZ
SUIT-2	G12D	HD	R273H	N/A	JCRB
PaTu 8988T	G12V	N/A	R282W	N/A	DSMZ
PANC1	G12D	HD	R273H	WT	ATCC
SW 1990	G12D	HD	P191del	WT	ATCC
HuP-T3	G12R	HD	R282W	WT	DSMZ
MIA PaCa-2	G12C	HD	R248W	WT	ATCC
KP-1N	G12D	N/A	N/A	WT	JCRB
BxPC3	WT	HD	Y220C	HD	ATCC

Gene Alteration: HD – Homozygous Deletion, INS – Insertion, N/A – Not Available, WT – Wild-Type; Source: DSMZ - Deutsche Sammlung von Mikroorganismen und Zellkulturen GmbH, ATCC – American Type Culture Collection, JCRB - Japanese Collection of Research Bioresources. Gene alteration data are compiled from multiple sources.

Supplementary Table 2: Antibodies used for western blotting

**Primary Antibodies**					
**Protein**	**Source**	**Catalog #**	**Isotype**	**Stock Concentration**	**Dilution**
α-Tubulin	Invitrogen	13-8000	Mouse	500 μg/ml	1:1000
AKT (pan)	Cell Signaling Technology	4691	Rabbit	N/A	1:1000
Beclin-1	Cell Signaling Technology	3495	Rabbit	N/A	1:1000
c-Myc	Cell Signaling Technology	5605	Rabbit	N/A	1:1000
Cleaved PARP	Cell Signaling Technology	5625	Rabbit	N/A	1:500
ERK1/2	Cell Signaling Technology	4695	Rabbit	N/A	1:1000
GAPDH	Thermo Fisher Scientific	MA5-15738	Mouse	1000 μg/ml	1:10000
GFP	Cell Signaling Technology	2956	Rabbit	N/A	1:1000
Hemagglutinin (HA) Tag	Bethyl Laboratories	A190-108A	Rabbit	1000 μg/ml	1:1000
KRAS	Calbiochem	OP24	Mouse	100 μg/ml	1:500
LATS1	Cell Signaling Technology	3477	Rabbit	N/A	1:1000
LATS2	Cell Signaling Technology	5888	Rabbit	N/A	1:500
LC3A/B	Cell Signaling Technology	12741	Rabbit	N/A	1:1000
MEK1/2	Cell Signaling Technology	8727	Rabbit	N/A	1:1000
Pan RAS	Calbiochem	OP40	Mouse	100 μg/ml	1:500
p21	Cell Signaling Technology	2947	Rabbit	N/A	1:1000
p62/SQSTM1	Santa Cruz Biotechnology	sc-28359	Mouse	200 μg/ml	1:1000
pAKT	Cell Signaling Technology	4058	Rabbit	N/A	1:1000
pERK1/2	Cell Signaling Technology	4370	Rabbit	N/A	1:1000
pMEK1/2	Cell Signaling Technology	9154	Rabbit	N/A	1:1000
pS6	Cell Signaling Technology	4858	Rabbit	N/A	1:1000
pYAP	Cell Signaling Technology	4911	Rabbit	N/A	1:1000
S6	Santa Cruz Biotechnology	sc-74459	Mouse	200 μg/ml	1:1000
STK38	Santa Cruz Biotechnology	sc-271703	Mouse	200 μg/ml	1:100
STK38L	A Hergovich - UCL	N/A	Rabbit	500 μg/ml	1:1000
YAP	Cell Signaling Technology	14074	Rabbit	N/A	1:1000
TAZ	Cell Signaling Technology	8418	Rabbit	N/A	1:1000
**Secondary Antibodies (HRP-Linked)**					
**Antibody**	**Source**	**Catalog #**	**Isotype**	**Concentration**	**Dilution**
Anti-Mouse IgG	Cell Signaling Technology	7076	N/A	N/A	1:5000
Anti-Rabbit IgG	Cell Signaling Technology	7074	N/A	N/A	1:5000

N/A – Not Available.

Supplementary Table 3: RT-qPCR Primer Sequences

Gene	Direction	Sequence
STK38	Forward	5′-TTTGGTGAGGTACGGCTTGT-3′
	Reverse	5′-ACTAGAATGTCACGCTCCGC-3′
STK38L	Forward	5′-GAGAGAGAAACCAGGCAGAAGA-3′
	Reverse	5′-GTCTGGTCCTTTTGAGCCGT-3′
LATS1	Forward	5′-TGTGGCCTATCATTCTGAGAGT-3′
	Reverse	5′-TCTGTCCGTTGCTAGGGTGA-3′
LATS2	Forward	5′-ACCCCAAAGTTCGGACCTTAT-3′
	Reverse	5′-GCATTTGCCGGTTCACTTCTG-3′
GAPDH	Forward	5′-GAGTCAACGGATTTGGTCGT-3′
	Reverse	5′-TTGATTTTGGAGGGATCTCG-3′

Supplementary Table 4: siRNA Target Sequences

Gene	siRNA	Target Sequence
STK38L	#1	5′-CUGGAGUUAAUAGAGUGAUUCAC-3′
	#2	5′-CACGCUCGCAAAGAAACAGAGUU-3′
CDKN1A/p21	#1	5′-CGACUGUGAUGCGCUAAUG-3′
	#2	5′-CCUAAUCCGCCCACAGGAA-3′
	#3	5′-CGUCAGAACCCAUGCGGCA-3′
	#4	5′-AGACCAGCAUGACAGAUUU-3′
LATS1	#1	5′-GGUGAAGUCUGUCUAGCAA-3′
	#2	5′-UAGCAUGGAUUUCAGUAAU-3′
	#3	5′-GGUAGUUCGUCUAUAUUAU-3′
	#4	5′-GAAUGGUACUGGACAAACU-3′
LATS2	#1	5′-GCACGCAUUUUACGAAUUC-3′
	#2	5′-ACACUCACCUCGCCCAAUA-3′
	#3	5′-AAUCAGAUAUUCCUUGUUG-3′
	#4	5′-GAAGUGAACCGGCAAAUGC-3′

Supplementary Table 5: PCR Primers for Gateway Cloning of HA-STK38L-PIF WT and HA-STK38L-PIF K119R

Primer	Sequence
Forward	5′-GGGGACAAGTTTGTACAAAAAAGCAGGCTTCGATATCACCATGGCCTACCCCTACG-3′
Reverse	5′-GGGGACCACTTTGTACAAGAAAGCTGGGTCGGATCCTCACCAGTCGGCGATGTAGT-3′

Supplementary Table 6: shRNA Target Sequences

Gene	Target Sequence
STK38L	5′-GAAGAAGGATTAGCAGATGAA-3′
YAP	5′-AAGCTTTGAGTTCTGACATCC-3′
TAZ	5′-GCGATGAATCAGCCTCTGAAT-3′
